# Leveraging the integration of bioinformatics and machine learning to uncover common biomarkers and molecular pathways underlying diabetes and nephrolithiasis

**DOI:** 10.3389/fimmu.2025.1574157

**Published:** 2025-07-11

**Authors:** Xudong Shen, Guoxiang Li, Junfeng Yao, Junping Yang, Xiaobo Ding, Zongyao Hao, Yan Chen, Yang Chen

**Affiliations:** ^1^ Department of Urology, The First Affiliated Hospital of Anhui Medical University, Hefei, China; ^2^ Institute of Urology, Anhui Medical University, Hefei, China; ^3^ Anhui Province Key Laboratory of Genitourinary Diseases, Anhui Medical University, Hefei, China; ^4^ Department of General Practice, Wuhu City Second People`s Hospital (Affiliated Wuhu Hospital of East China Normal University)Wuhu, Anhui, China

**Keywords:** kidney stone, diabetes, machine learning, bioinformatics, programmed cell death

## Abstract

**Background:**

Kidney stones are a common benign condition of the urinary system, characterized by high incidence and recurrence rates. Our previous studies revealed an increased prevalence of kidney stones among diabetic patients, suggesting potential underlying mechanisms linking these two conditions. This study aims to identify key genes, pathways, and immune cells that may connect diabetes and kidney stones.

**Methods:**

We conducted bulk transcriptome differential analysis using our sequencing data, in conjunction with the AS dataset (GSE231569). After eliminating batch effects, we performed differential expression analysis and applied weighted gene co-expression network analysis (WGCNA) to investigate associations with 18 forms of cell death. Differentially expressed genes (DEGs) were subsequently analyzed using 10 commonly used machine learning algorithms, generating 101 unique combinations to identify the final DEGs. Functional enrichment analysis was performed, alongside the construction of protein-protein interaction (PPI) networks and transcription factor (TF)-gene interaction networks.

**Results:**

For the first time, bioinformatics tools were utilized to investigate the close genetic relationship between diabetes and kidney stones. Among 101 machine learning models, S100A4, ARPC1B, and CEBPD were identified as the most significant interacting genes linking diabetes and kidney stones. The diagnostic potential of these biomarkers was validated in both training and test datasets.

**Conclusion:**

We identified three biomarkers—S100A4, ARPC1B, and CEBPD—that may play critical roles in the shared pathogenesis of diabetes and kidney stones. These findings open new avenues for the diagnosis and treatment of these comorbid conditions.

## 1 Introduction

Urinary system stones, also known as urolithiasis, are caused by the abnormal accumulation of crystalline substances in the kidneys (1). Recent evidence suggests that the global prevalence of urolithiasis has been rising in recent years due to various factors, including societal changes, dietary habits, climate variations, and comorbidities (2, 3). Studies have indicated that kidney stones are not merely a result of urinary metabolic abnormalities but are also associated with multiple metabolic features such as central obesity, elevated triglyceride levels, hypertension, and diabetes (4). In our previous research, we identified insulin resistance as a central factor in the pathogenesis of diabetic kidney stones (5). Insulin resistance may lower urinary pH by disrupting ammonium production and enhancing the reabsorption of sodium and bicarbonate (6). Additionally, compensatory hyperinsulinemia due to insulin resistance may increase urinary calcium excretion, thereby promoting the formation of calcium-based kidney stones in diabetic patients (7, 8). However, the underlying mechanisms linking diabetes and kidney stones remain poorly understood. Therefore, elucidating the distinct mechanisms driving the progression of these two diseases is of paramount importance.

Cell death is a fundamental biological process associated with various life phenomena, including growth, development, aging, and disease (9). Based on triggering mechanisms, cell death can be categorized into programmed cell death (PCD) and accidental cell death (ACD). PCD is a controlled and sequential process regulated by specific molecular mechanisms, and it can be modulated through pharmacological or genetic interventions. PCD occurs via diverse mechanisms, including apoptosis, autophagy, pyroptosis, ferroptosis, necroptosis, and NETosis, among others. Notably, many nephroprotective mechanisms of drugs are based on modulating PCD. For instance, irbesartan and metformin alleviate renal apoptosis induced by advanced glycation end products (AGEs) (10, 11), while fenofibrate and sodium-glucose cotransporter 2 (SGLT2) inhibitors enhance autophagy to improve kidney function in patients with diabetic kidney disease (DKD) (12, 13). Additionally, SGLT2 inhibition has been reported to suppress kidney stone formation (14).

Nevertheless, the role of PCD in the biological mechanisms underlying kidney stone and diabetes pathogenesis remains incompletely understood. To clarify the shared biomarkers and PCD pathways involved in these two conditions, we first identified these biomarkers using microarray and RNA sequencing datasets. A variety of bioinformatics analyses were subsequently employed to uncover key biomarkers and biological functions, providing valuable perspectives on potential innovative therapeutic targets.

## 2 Methods

### 2.1 Mouse models of diabetes and diabetic kidney stones

All experimental procedures were conducted in compliance with the guidelines of the National Institutes of Health for the Care and Use of Laboratory Animals. Male C57BL/6J mice (6–8 weeks old) were used in the study. Diabetes was induced by intraperitoneal injection of streptozotocin (STZ) at 40 mg/kg for one week. After one week, blood glucose levels were measured using the OneTouch Ultra Glucometer (LifeScan, Milpitas, CA, USA) via the glucose oxidase method. Mice with blood glucose levels >16.7 mmol/L were selected for further studies. To establish a kidney stone model, the mice were administered glyoxylate (GA, 75 mg/kg, 200 μL) daily for one week. One week later, mice were sacrificed following standard experimental procedures. Kidney sections were stained with hematoxylin and eosin (H&E) and examined under a polarized microscope (Zeiss, Germany). The presence of crystalline deposits in the kidneys confirmed the successful establishment of the kidney stone model. After model validation, RNA sequencing was performed for subsequent analyses.

### 2.2 Sample sources

In addition to RNA sequencing data from mouse models, microarray datasets from the Gene Expression Omnibus (GEO) database were analyzed, including GSE231569 and GSE73680. The GSE231569 dataset comprised four renal papilla samples and one renal medulla sample. The GSE73680 dataset included 29 kidney stone patient samples and 33 normal samples.

### 2.3 Differential expression analysis

PCD-related genes were sourced from the literature (9). The preprocessCore R package was used to normalize the data, and differential expression analysis of PCD-related genes was conducted using the limma R package. Differentially expressed genes (DEGs) were identified based on the criteria of adjusted P.Val < 0.05 and |logFC| > 0.25. These DEGs were used in subsequent analyses to identify genes with altered expression between disease and normal samples.

### 2.4 Weighted gene co-expression network analysis

WGCNA (15) was performed on self-generated datasets to identify gene modules associated with kidney stones and diabetes. Prior to clustering, missing data points were evaluated. A soft-threshold power (β) was selected based on the scale-free topology standard to construct a biologically meaningful network. A topological overlap matrix (TOM) was derived from the adjacency matrix, and gene modules were identified using the dynamic tree-cutting algorithm. Gene significance (GS), module membership (MM), and correlations between modules and clinical traits were calculated. Pearson correlation coefficients and p-values of module eigengenes with disease traits facilitated the identification of key modules related to kidney stones and diabetes. Key genes from these modules were integrated, and shared genes were identified using the AWFE diagram.

### 2.5 Enrichment analysis

Functional enrichment analysis was conducted on DEGs. Gene Ontology (GO) and Kyoto Encyclopedia of Genes and Genomes (KEGG) pathway analyses were performed using the R packages “org.Hs.eg.db,” “ggplot2,” “clusterProfiler,” and “enrichplot.” GO analysis identified biological processes (BP), molecular functions (MF), and cellular components (CC) associated with overlapping genes, while KEGG analysis identified enriched signaling pathways. Keywords were clustered based on similarity, with the most enriched and representative terms selected. Statistical significance was determined with a threshold of p < 0.05.

### 2.6 Single-cell RNA data processing

The single-cell RNA sequencing (scRNA-seq) dataset GSE231569 was reanalyzed. Data filtering and analysis were performed using the Seurat R package. ScRNA-seq data were filtered with thresholds of nFeature_RNA < 5000, percent_mito < 15, percent_ribo > 3, and percent_hb < 0.1. Cell clustering results were visualized using TSNE and UMAP methods. Cell types were annotated with the SingleR package (version 2.4.1). WGCNA (version 1.72-5) identified DEGs in the GSE231569 dataset. Cluster-specific DEGs were identified using the FindAllMarkers function with a resolution of 0.8. DEGs from scRNA-seq data (using Seurat) and bulk RNA-seq data (using limma) were intersected with WGCNA and PCD-related genes, yielding 12 potential prognostic genes. Protein-protein interaction (PPI) networks were visualized using STRING (https://string-db.org) and GeneMANIA (http://genemania.org).

### 2.7 Machine learning to identify key PCD genes

To pinpoint key PCD genes associated with diabetes and kidney stones, 10 machine learning algorithms were applied, generating 101 unique combinations (16–18) (PMIDs: 33794304, 35145098, 39346927). Model performance was evaluated using an independent validation set (GSE73680). The glmBoost+RF combination performed best, ultimately identifying three key genes. Correlation analysis of these genes with all other genes was visualized using heatmaps displaying the top 50 positively correlated genes. Gene set enrichment analysis (GSEA) was performed to explore functional correlations of the key genes. Upstream transcription factors were predicted using the RegNetwork database (https://regnetworkweb.org), and networks were constructed with Cytoscape software.

### 2.8 Western blotting

Protein extraction was performed using RIPA lysis buffer (Solarbio, China) with protease inhibitors. Protein concentration was quantified using a BCA protein assay kit (Biosharp, China). Equal amounts of total protein (30 μg) were separated by 10% SDS-PAGE and transferred to NC membranes (Cytiva, China). Membranes were blocked with 5% non-fat milk for 1 hour at room temperature and incubated overnight at 4°C with primary antibodies against ARPC1B, S100A4, CEBPD, and β-actin (Affinity, China). After incubation with secondary antibodies for 1 hour, protein bands were visualized using enhanced chemiluminescence (Tanon, China) and captured with a Tanon imaging system.

### 2.9 Real-time PCR analysis

Total RNA was extracted using TRIzol and quantified with a NanoDrop 2000 (Thermo Fisher Scientific, Wilmington, DE, USA). Reverse transcription was performed using ToloScript All-in-one RT EasyMix for qPCR. Real-time quantitative PCR was conducted with Q3 SYBR qPCR Master Mix on an ABI 7500 system to evaluate RNA expression levels. Primer sequences were as follows:

S100A4: Forward: 5’-TGAGCAACTTGGACAGCAACA-3’, Reverse: 5’-CTTCTTCCGGGGCTCCTTTATC-3’;

ARPC1B: Forward: 5’-GGAACAAGGACCGTACACAGA-3’, Reverse: 5’-CAATGCGGTTACTCTCAGGGG-3’;

CEBPD: Forward: 5’-CAAGAACAGCAACGAGTACCG-3’, Reverse: 5’-GTCACTGGTCAACTCCAGCAC-3’.

### 2.10 Statistical analysis

Data analysis was conducted using GraphPad Prism 9.0 software. Results are presented as mean ± SEM. Unpaired t-tests were used for two-group comparisons, while one-way or two-way ANOVA (with Dunnett or Tukey multiple comparison tests) was applied for comparisons among three or more groups. Log-rank (Mantel-Cox) tests were used for survival analysis. A p-value < 0.05 was considered statistically significant.

## 3 Results

### 3.1 Single-cell transcriptional profiling in kidney stones based on PCD

To elucidate the potential processes of PCD (programmed cell death) during kidney stone formation, we reanalyzed a previously published scRNA-seq dataset (GSE231569) using the Seurat method. After quality control filtering (**Supplementary Figure 1**), a total of 21,432 unique genes were identified from 29,609 cells across four samples. Annotation of these genes identified eight distinct cell clusters (**Supplementary Figure 2**). Using the FindAllMarkers function, we identified differentially expressed genes (DEGs) for each cell type, showcasing the top five genes per cell type (**Supplementary Figure 2B**). Marker genes were analyzed using the COSG R package, with the top 10 genes visualized in a heatmap (**Supplementary Figure 2C**). Additionally, KEGG and Reactome analyses were performed for the top 100 marker genes per cell type, displaying the top five enriched pathways for each (**Supplementary Figures 2D, E**).

**Figure 1:**
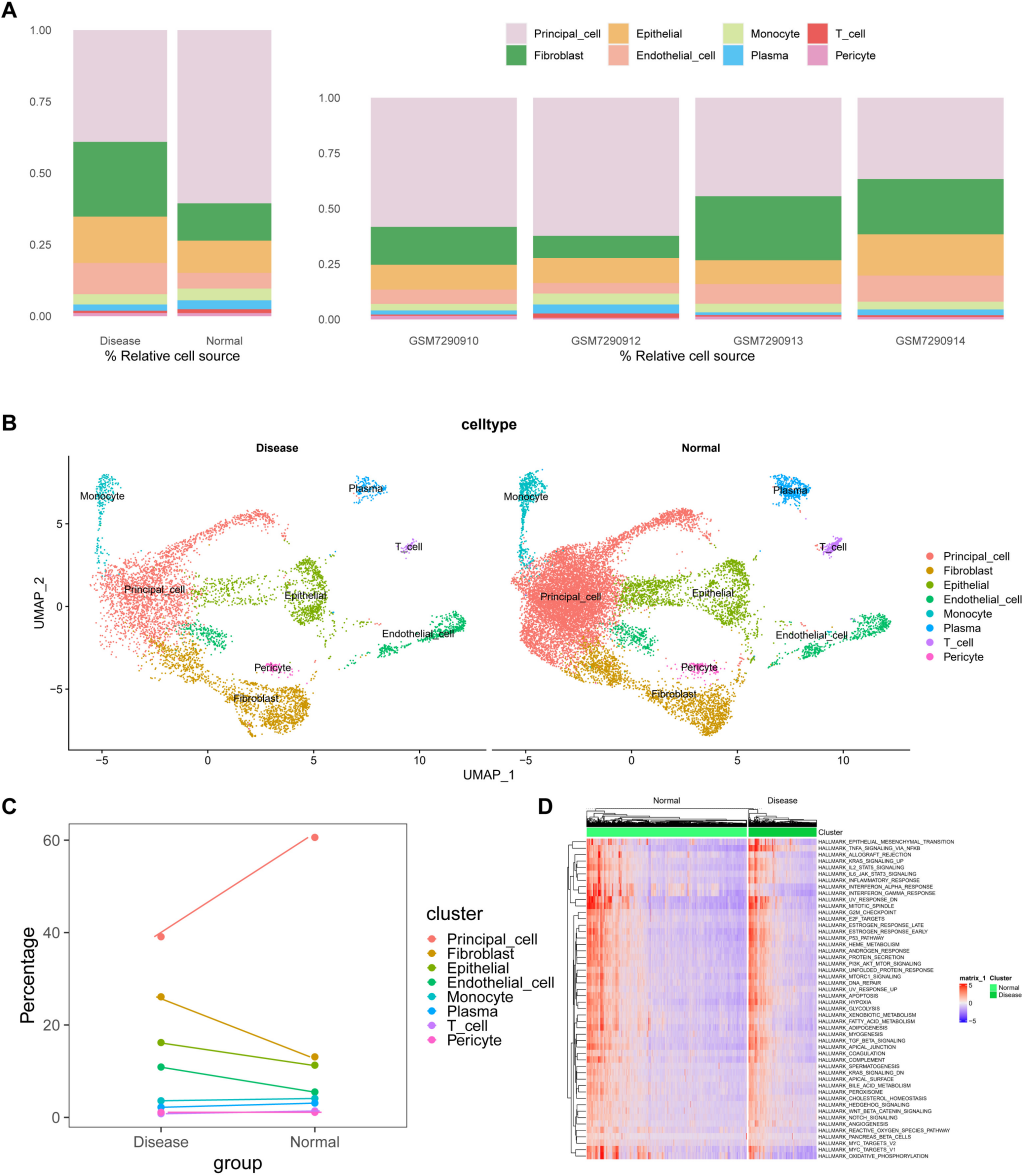
scRNA-seq analyses and cluster annotation of renal tissues in kidney stone patients. **(A)**. Stacked bar graph showing the proportions of different cell types (Fibroblast, Epithelial, Endothelial, Monocyte, Plasma cell, T cell, Pericyte, and Principal cell) identified from the GSE231569 dataset based on canonical marker genes. **(B)**. UMAP-based dimensionality reduction for cell clustering and visualization, with cells color-coded by cluster annotation. **(C)**. Dynamic changes in cell-type composition across samples, highlighting the enrichment of fibroblasts, epithelial, and endothelial cells in kidney stone tissues. **(D)**. GSVA-based pathway enrichment heatmap for hallmark gene sets from the MSigDB database, demonstrating the activation (red) or inhibition (green) of pathways in different samples. Statistical differences between groups were calculated using Student’s t-test or the Kruskal-Wallis test. These analyses reveal the heterogeneity of the cellular and transcriptional landscape in nephrolithiasis.

**Figure 2:**
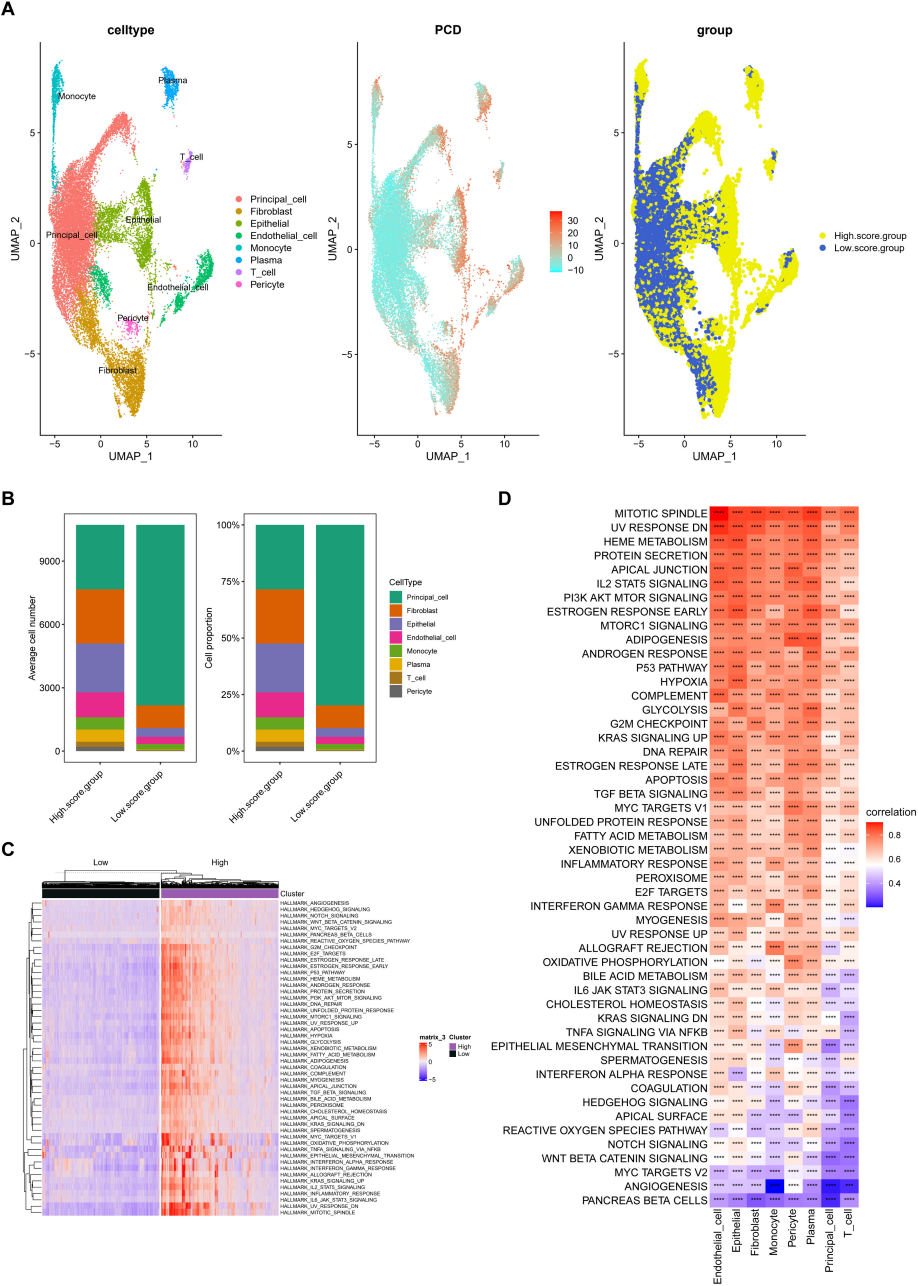
Relationship between kidney stone–derived cells and programmed cell death (PCD). **(A)**. UMAP visualization of PCD gene set enrichment scores across individual cells using GSVA, dividing cells into high and low PCD score groups. **(B)**. Cell type proportions in different PCD score groups, showing increased frequency of Fibroblasts, Epithelial cells, Endothelial cells, and immune cells in high-score clusters. **(C, D)**. Correlation between PCD scores and hallmark pathway activities within each cell type, suggesting biological pathways associated with high cell death signatures. These findings link cell fate decisions with nephrolithiasis-associated inflammation and remodeling. ****p < 0.0001.

After normalizing gene expression data, we conducted PCA and UMAP-based clustering in the PCA space to identify cell types. These clusters were annotated based on marker genes into recognizable cell types: Fibroblast, Epithelial, Endothelial_cell, Monocyte, Plasma, T_cell, Pericyte, and Principal_cell (**Figure 1**). **Figures 1B, C** depict the proportions of different cell types across samples, highlighting an enrichment of Fibroblast, Epithelial, and Endothelial cells in kidney stone patients. Using the GSVA R package, we scored each cell type for hallmark pathways downloaded from the MSigDB database (https://www.gsea-msigdb.org/gsea/msigdb). Heatmaps of hallmark pathway scores revealed pathways like “EPITHELIAL_MESENCHYMAL_TRANSITION” and “TNFA_SIGNALING_VIA_NFKB” as prominent (**Figure 1D**). Next, we investigated the relationship between cells from kidney stone samples and PCD. PCD gene sets were scored using GSVA, and UMAP visualized the PCD gene set scores, grouping cells into high and low score categories (**Figure 2A**). As shown in **Figure 2B**, high PCD score groups exhibited significantly increased proportions of Fibroblast, Epithelial, Endothelial_cell, Monocyte, Plasma, T_cell, and Pericyte cells, with a concurrent reduction in Principal cells. The relationship between hallmark pathway scores and PCD scores was further explored for each cell type (**Figure 2C, D**).

### 3.2 Establishing diabetic and diabetic nephrolithiasis mouse models

The aim of this study was to investigate the role of PCD genes in nephrolithiasis and diabetes. However, no existing datasets combine sequencing data for these two conditions. To address this, we conducted relevant animal experiments to obtain diabetic and diabetic nephrolithiasis samples for transcriptomic analysis.

**Figure 3:**
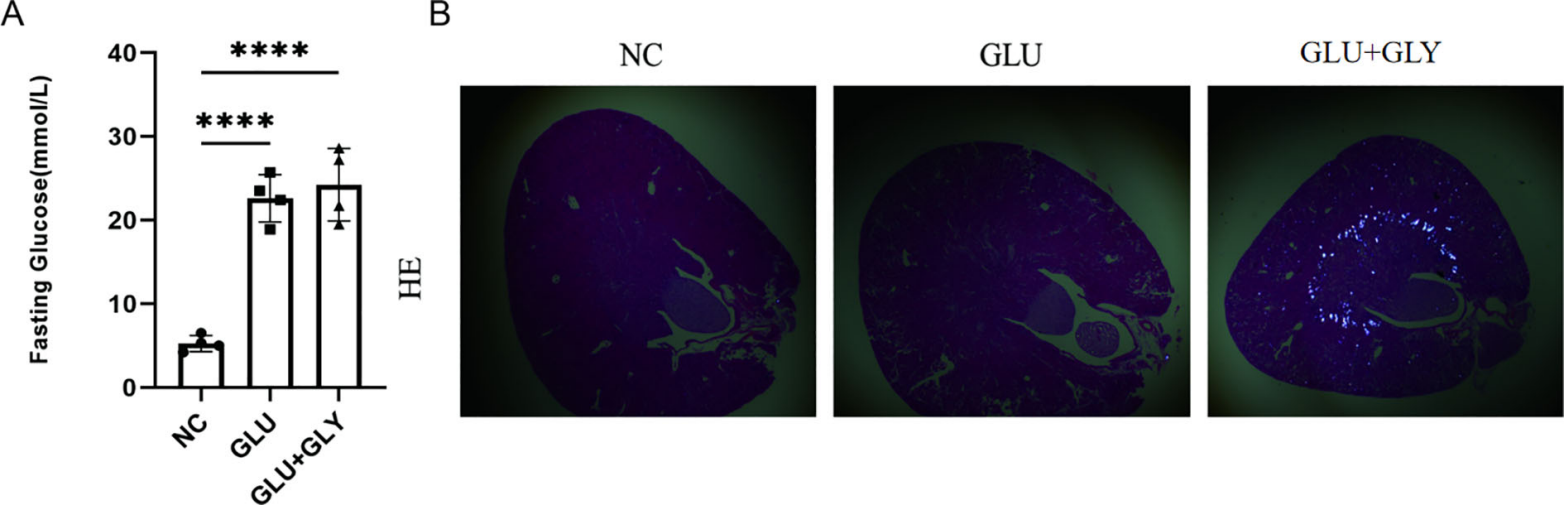
Construction and validation of diabetic and diabetic nephrolithiasis mouse models. **(A)**. Blood glucose levels in different mouse groups (Control, STZ-induced diabetes, and STZ+GA-induced diabetic nephrolithiasis), confirming successful establishment of the hyperglycemic model (P < 0.001). **(B)**. Representative hematoxylin and eosin (H&E) staining images under polarized light microscopy showing calcium oxalate crystal deposits in the kidneys of the STZ+GA group. These findings verify the *in vivo* modeling of combined diabetes and nephrolithiasis. ****p < 0.0001.

First, we established animal models. Blood glucose levels were significantly elevated in the STZ and STZ+GA groups compared to the control group (P < 0.001), with no statistically significant difference between the STZ and STZ+GA groups (**Figure 3A**). Under polarized light microscopy, the STZ+GA group showed evident crystal deposits compared to the STZ group (**Figure 3B**). These results confirm the successful establishment of the diabetic and diabetic nephrolithiasis mouse models.

### 3.3 Identification and functional enrichment analysis of DEGs in diabetes and nephrolithiasis

**Figure 4:**
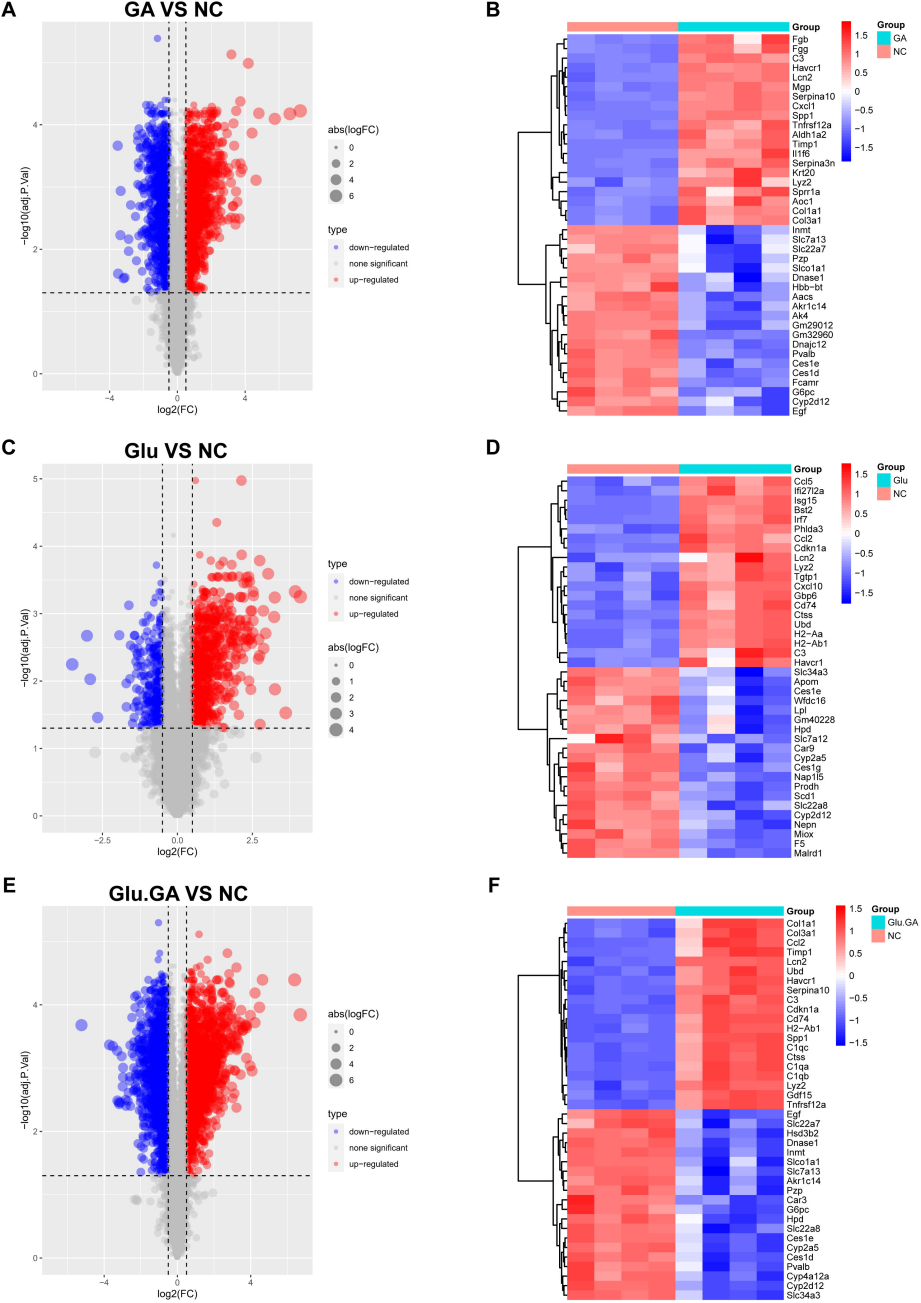
Identification and visualization of differentially expressed genes (DEGs). **(A, C, E)**. Volcano plots showing DEGs between disease and control groups (GLU vs. NC, GA vs. NC, and GLU+GA vs. NC), with color-coded significance thresholds (adjusted P < 0.05, |logFC| > 0.25). **(B, D, F)**. Heatmaps displaying the top 40 DEGs by variance across samples, clustering genes and conditions to illustrate transcriptomic divergence. These results provide a basis for identifying shared gene signatures between diabetes and nephrolithiasis.

To investigate the molecular interactions between diabetes and nephrolithiasis, mice were divided into four groups for transcriptomic sequencing: normal control (NC), STZ (GLU), GA, and STZ+GA (GLU+GA). Differentially expressed genes (DEGs) were identified using the “limma” package in R, comparing GLU vs. NC, GA vs. NC, and GLU+GA vs. NC. Volcano plots were generated to visualize the results (**Figures 4A, C, E**).

**Figure 5:**
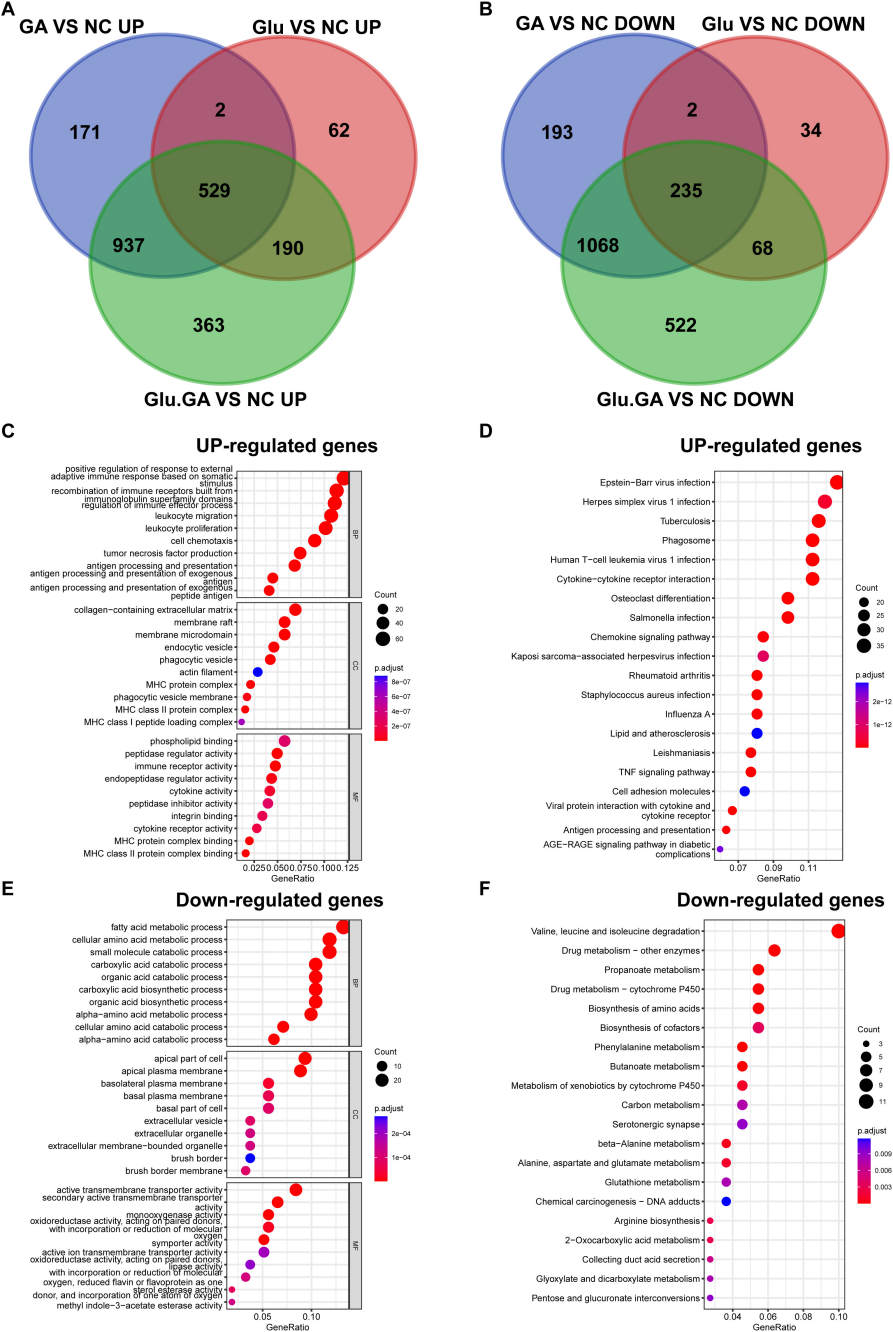
Identification of common DEGs and functional enrichment analysis. **(A, B)**. Venn diagrams showing the overlap of DEGs from the diabetes and nephrolithiasis groups, revealing 764 shared genes (529 upregulated, 235 downregulated). **(C)**. GO enrichment analysis of upregulated genes indicating involvement in immune activation, extracellular matrix organization, and cellular signaling. **(D)**. KEGG pathways of upregulated genes highlighting virus infection-related pathways, possibly linked to innate immune activation. **(E, F)**. GO and KEGG analysis of downregulated genes associated with amino acid metabolism, transmembrane transport, and drug metabolism.

To further explore the most significant differences between the disease and control groups, heatmaps highlighting the top 40 most variable genes were constructed (**Figures 4B, D, F**). By comparing the genes present in both diseases, a Venn diagram revealed a total of 764 shared differentially expressed genes (DEGs), with 529 genes upregulated and 235 genes downregulated (**Figures 5A, B**). Gene Ontology (GO) and Kyoto Encyclopedia of Genes and Genomes (KEGG) analyses were performed to determine the potential underlying mechanisms.The GO analysis revealed that the upregulated genes were primarily associated with biological processes (BP) such as “positive regulation of response to external stimulus,” “adaptive immune response based on somatic recombination of immune receptors built from immunoglobulin superfamily domains,” and “regulation of immune effector process.” In cellular components (CC), the upregulated genes were mainly localized to the “collagen-containing extracellular matrix,” “membrane raft,” and “membrane microdomain.” In molecular functions (MF), the upregulated genes significantly contributed to “phospholipid binding,” “actin binding,” and “peptidase regulator activity.” These findings suggest that upregulated DEGs play a role in maintaining cell structure, cell homeostasis, cell communication, and fundamental cell growth (**Figure 5C**).

KEGG pathway analysis emphasized that upregulated DEGs were significantly enriched in pathways such as “Epstein-Barr virus infection” and “Herpes simplex virus 1 infection” (**Figure 5D**). For the downregulated DEGs, GO analysis indicated that they were mainly related to biological processes such as “fatty acid metabolic process,” “cellular amino acid metabolic process,” and “small molecule catabolic process.” In cellular components (CC), the downregulated DEGs were primarily localized to the “apical part of cell,” “apical plasma membrane,” and “basolateral plasma membrane.” In molecular functions (MF), downregulated DEGs contributed significantly to “active transmembrane transporter activity,” “secondary active transmembrane transporter activity,” and “monooxygenase activity” (**Figure 5E**). KEGG pathway analysis highlighted significant enrichment of downregulated DEGs in pathways such as “Valine, leucine, and isoleucine degradation” and “Drug metabolism - other enzymes” (**Figure 5F**).

### 3.4 WGCNA identified key modules in kidney stones and diabetes

**Figure 6:**
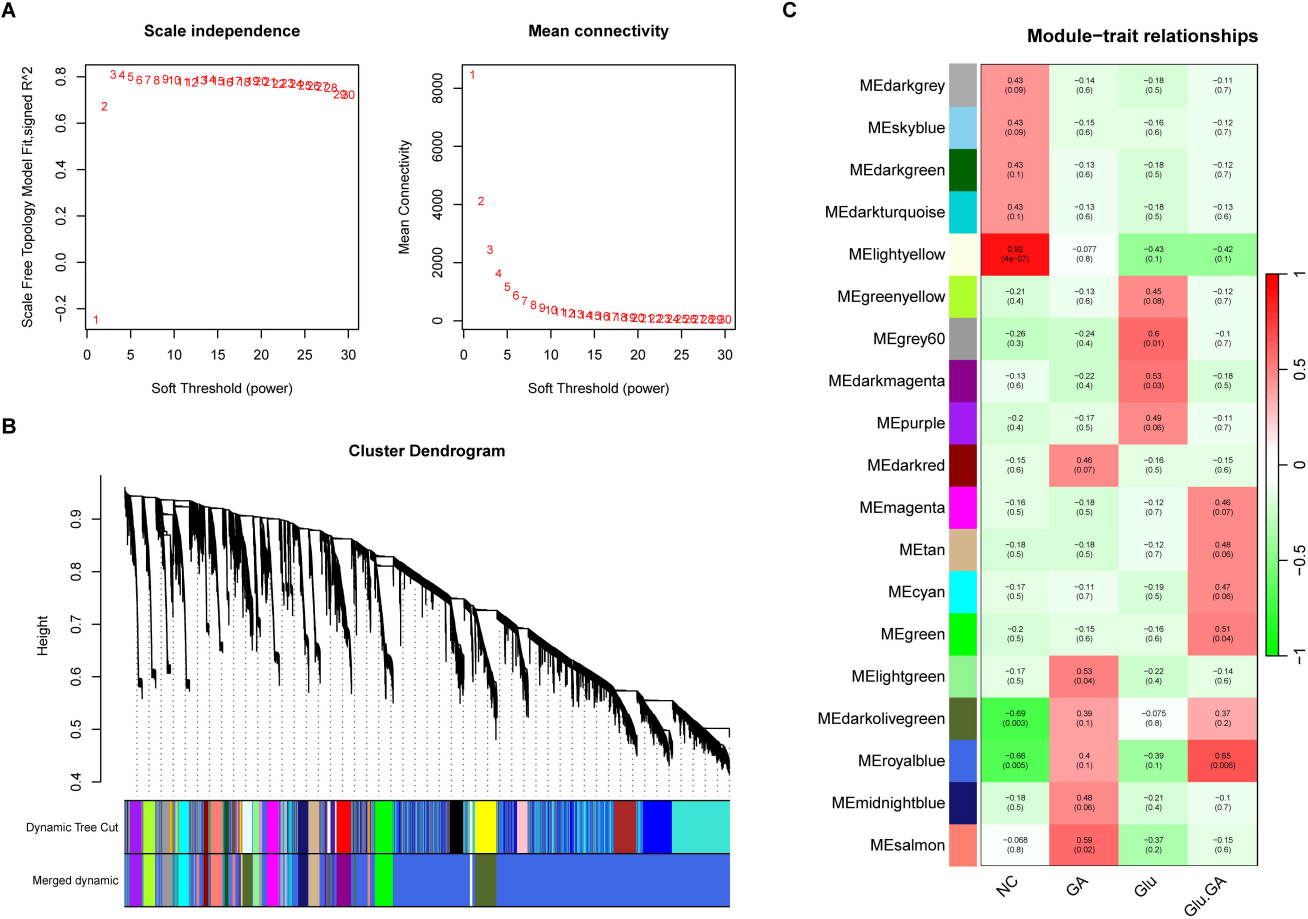
Weighted gene co-expression network analysis (WGCNA) to identify trait-related modules. **(A)**. Determination of optimal soft-thresholding power to ensure scale-free topology in the co-expression network. **(B)**. Hierarchical clustering dendrogram showing 19 distinct gene modules, each assigned a unique color. **(C)**. Heatmap correlating module eigengenes with clinical traits (e.g., diabetes, nephrolithiasis), identifying four key modules (lightyellow, green, darkolivegreen, and royalblue) significantly associated with disease status.

WGCNA revealed 19 relevant modules in the self-test data, each represented by a unique color. A module-trait relationship heatmap was generated to assess the association of each module with the disease (**Figures 6B, C**). The WGCNA gene selection showed that NC or Glu+GA modules differed from the other three groups, with statistically significant differences. The results indicated that the following modules were significantly correlated with the disease:MElightyellow (|r| = 0.92, p < 0.0001),MEgreen (|r| = 0.51, p = 0.04),MEdarkolivegreen (|r| = -0.69, p = 0.003),MEroyalblue (|r| = -0.66, p = 0.005)These four modules together contained a total of 28,162 genes.

### 3.5 Identification of Key PCD genes in kidney stones and diabetes and enrichment analysis

**Figure 7:**
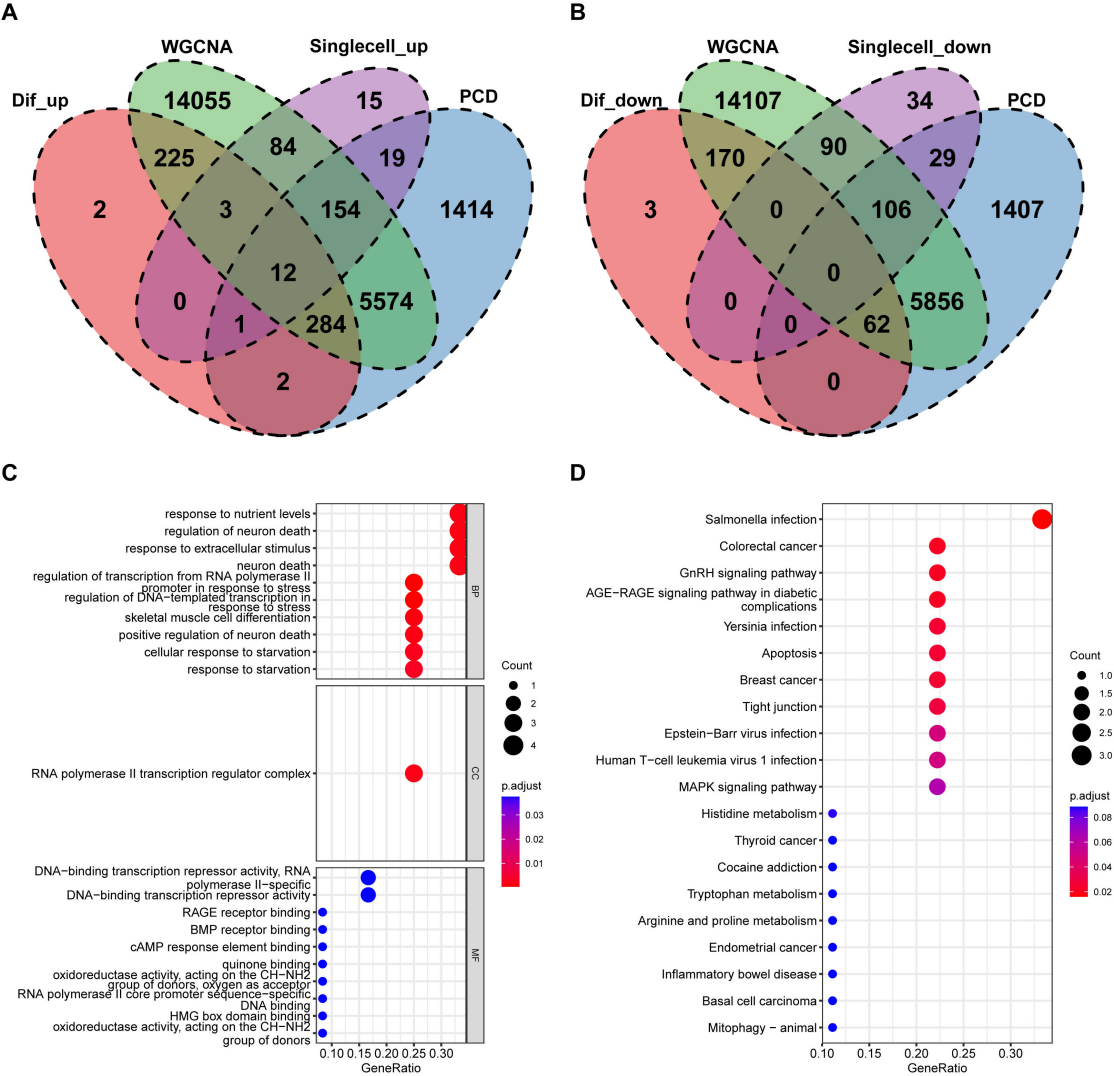
Identification of key PCD genes by integrated analysis and functional annotation. **(A, B)**. Venn diagrams integrating DEGs from bulk RNA-seq, scRNA-seq, WGCNA modules, and curated PCD gene lists, identifying 12 upregulated candidate genes (e.g., S100A4, ARPC1B, CEBPD). **(C, D)**. GO and KEGG pathway enrichment analysis of the 12 intersected genes, highlighting biological processes related to nutrient sensing, apoptosis, and immune signaling.

Based on the previous results, the intersection of single-cell differential analysis genes, bulk differential genes, WGCNA genes, and PCD genes was obtained, including 12 upregulated genes: EGR1, ATF3, AOC1, BTG2, JUN, RHOB, S100A4, GDF15, GADD45B, CEBPD, DDIT4, and ARPC1B. No downregulated genes were identified (**Figures 7A, B**). Further GO analysis revealed that these genes were mainly involved in biological processes (BP) such as “response to nutrient levels,” “regulation of neuron death,” and “response to extracellular stimulus.” In terms of cellular components (CC), the shared PCD genes were predominantly localized to the “RNA polymerase II transcription regulator complex.” For molecular functions (MF), the shared PCD genes significantly contributed to “DNA-binding transcription repressor activity.”KEGG pathway analysis emphasized the significant enrichment of shared PCD genes in pathways such as “Salmonella infection” (**Figures 7C, D**). Next, we analyzed the differences in the expression levels of the 12 genes between the GLU+GA group and the NC group. The results indicated that the expression levels of all 12 genes were significantly higher in the GLU+GA group compared to the NC group (**Supplementary Figure 3**). Finally, PPI networks were constructed using STRING and GENEMANIA databases (**Supplementary Figure 4**).

### 3.6 Identification of candidate diagnostic biomarkers for kidney stones in diabetic patients using machine learning algorithms

**Figure 8:**
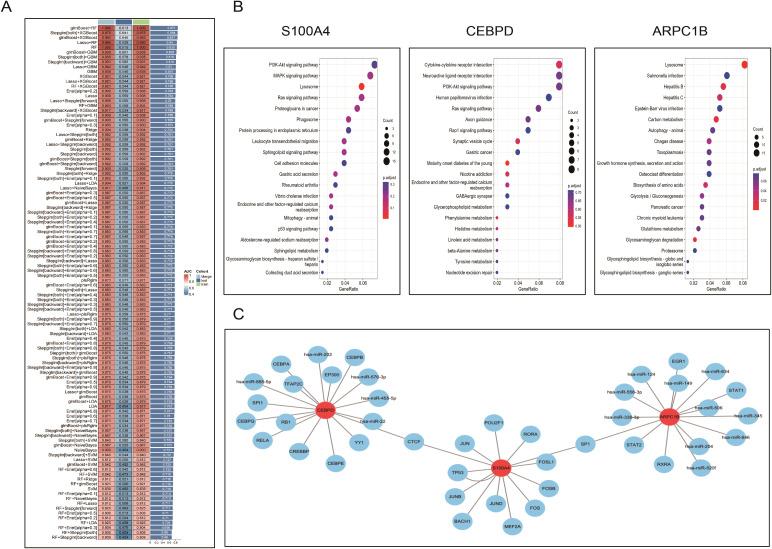
Machine learning–based identification and characterization of diagnostic biomarkers. **(A)**. Performance evaluation of 101 machine learning algorithm combinations to build prognostic models; the glmBoost+RF combination demonstrated optimal AUC and stability. **(B)**. Heatmaps showing genes most positively correlated with the three hub genes (S100A4, ARPC1B, CEBPD), based on co-expression analysis. **(C)**. GSEA analysis of the hub genes identifying enriched pathways involving inflammation, oxidative stress, cell cycle, and apoptosis. **(D)**. Transcription factor–gene regulatory network predicted via the RegNetwork database, visualized in Cytoscape.

Based on the previous results, we obtained 12 PCD genes. In this study, we employed machine learning to construct prognostic models from these genes. Using the GEO dataset, we built 101 prognostic models and evaluated the performance of each model in an independent validation set (GSE73680). The results indicated that the glmBoost+RF algorithm performed the best, ultimately identifying three key genes: S100A4, ARPC1B, and CEBPD (**Figure 8A**; **Supplementary Table 1**).Next, we performed correlation analysis of these three genes with all other genes and displayed the expression of the top 50 positively correlated genes using heatmaps (**Supplementary Figure 5**). We also investigated the signaling pathways enriched by these three feature genes to explore their potential molecular mechanisms in the progression of diabetes and kidney stones. **Figure 8C** lists the top 20 enriched pathways from GSEA analysis, showing that the feature genes play a crucial role in regulating inflammation, cell metabolism, apoptosis, and the cell cycle in the progression of diabetes and kidney stones.Finally, we used the RegNetwork database (https://regnetworkweb.org/) to predict transcription factors upstream of these genes. The core genes are highlighted in red, and only genes present in the database are shown. A PPI network was constructed using Cytoscape software (**Figure 8**).

### 3.7 Expression of biomarkers and western blot validation in animal models

**Figure 9:**
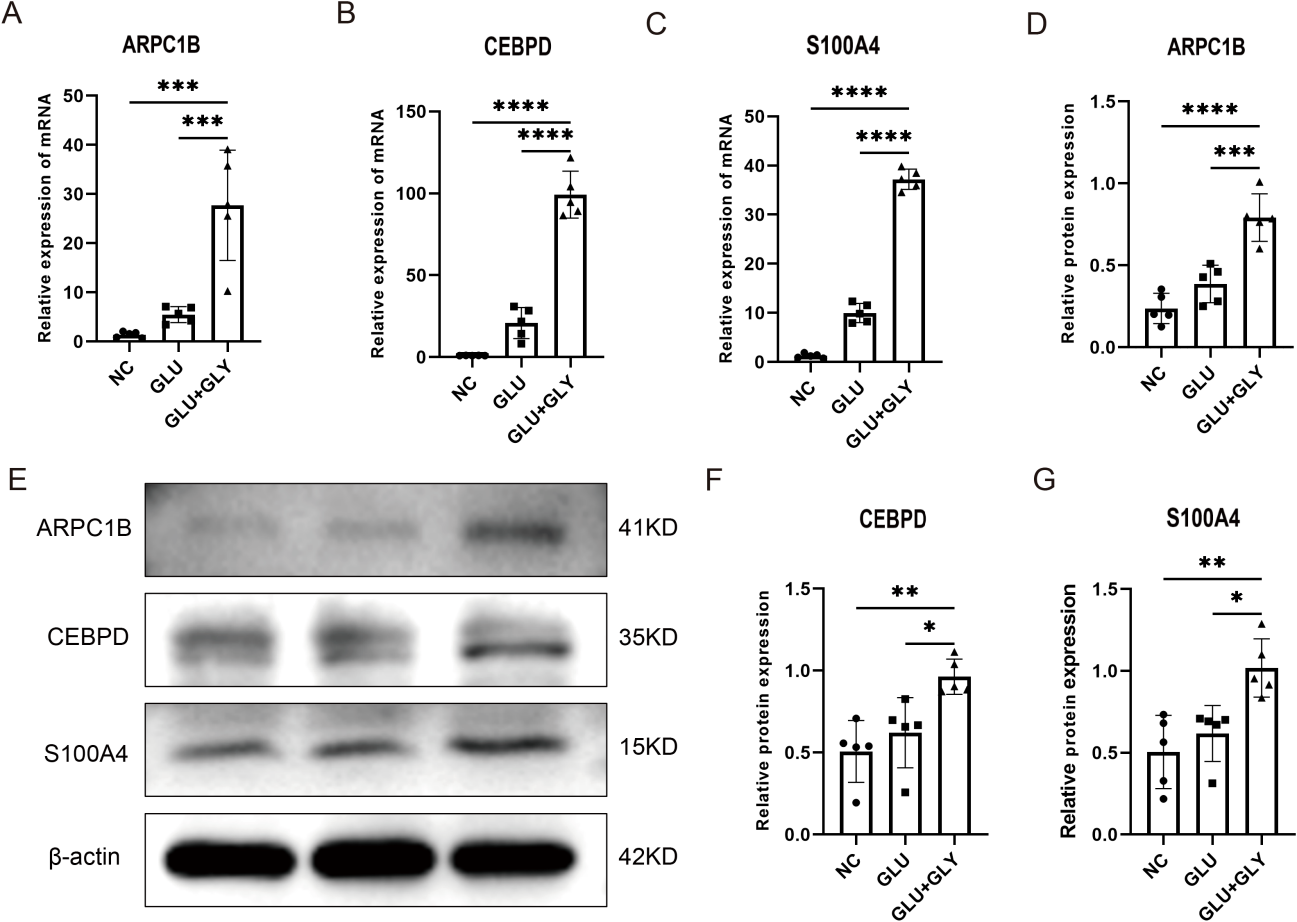
Experimental validation of biomarker expression in animal models. **(A–C)**. Quantitative PCR analysis showing significantly elevated expression of S100A4, ARPC1B, and CEBPD in diabetic nephrolithiasis mouse models compared to controls. **(D–G)**. Western blot analysis further confirming increased protein expression of the three biomarkers. Densitometry quantification supports the transcriptomic findings. *p < 0.05, **p < 0.01, ***p < 0.001, ****p < 0.0001.

We validated the mRNA expression of these key genes in diabetic and kidney stone comorbidity mice. We found that after kidney stone treatment, the expression of S100A4, ARPC1B, and CEBPD genes in diabetic mice was significantly elevated. Western blotting further confirmed that the protein levels of S100A4, ARPC1B, and CEBPD were significantly upregulated.These results collectively suggest that S100A4, ARPC1B, and CEBPD may serve as core biomarkers in the common mechanisms underlying diabetes and kidney stones (**Figure 9**).

## 4 Discussion

Kidney stones and diabetes are prevalent clinical issues that severely impact patients’ quality of life. Previous epidemiological studies have confirmed that diabetes increases the incidence of kidney stones (5). Unraveling the potential mechanisms underlying the interaction between diabetes and kidney stones could inspire novel therapies for both conditions, paving the way for beneficial treatments. This study uses bioinformatics analysis to deeply explore the molecular features of common pathways in diabetes and kidney stones, and based on machine learning, identifies three novel biomarkers related to the diagnosis and treatment of these conditions.

In this study, we initiated our analysis by obtaining data from the GEO database, specifically the GSE231569 dataset (patients diagnosed with kidney stones). Using DEGs and PCD, we applied GSVA to score the PCD gene set. In the high PCD score group, we observed a marked increase in the content of fibroblast, epithelial, endothelial cells, monocytes, plasma cells, T cells, and pericytes. Further pathway analysis revealed significant associations between the high-score group and pathways such as the PI3K-Akt signaling pathway, inflammation response, and metabolic processes. The PI3K-Akt pathway is essential for cellular functions like cell cycle regulation, proliferation, metabolism, survival, growth, and angiogenesis, and is considered crucial in the pathogenesis of both diabetes and kidney stones (19, 20). To further elucidate the main molecular clusters common to both diabetes and kidney stones, we conducted a multi-omics study integrating single-cell data with machine learning. Comprehensive analysis of multi-omics data helps us better understand the disease-specific regulatory mechanisms (21). The selection of common omics clustering methods is often influenced by personal preference, which further broadens the limitations of specific methods (22). Machine learning algorithms have emerged to address these shortcomings, and they have become an effective means of analyzing multi-omics data (17). In this study, we integrated the latest 10 clustering algorithms, creating 101 algorithm combinations to identify the best model and overcome the limitations of algorithm selection. At present, when artificial intelligence algorithms are combined with large biological datasets, overfitting is an important issue during model construction (23). Models that perform well in training datasets often fail to generalize effectively to independent validation sets. To avoid overfitting issues in the training cohort, we used the AUC index of the validation cohort as the sorting criterion and selected the glmBoost+RF algorithm, which demonstrated the best performance.

One of the strengths of this study is the identification of three novel biomarkers for diabetes and kidney stones using machine learning methods: S100A4, ARPC1B, and CEBPD. S100A4, also known as metastasis-associated protein (Mst1) or fibroblast-specific protein (FSP1), is a member of the S100 calcium-binding protein family (24). It is highly expressed in tumor cells and is closely related to cancer proliferation, invasion, and metastasis (25). Recently, S100A4 has been implicated in the progression of fibrosis in various organs (26–29) and in inflammatory diseases such as rheumatoid arthritis (30–32). A common feature of these pathological conditions is the involvement of inflammation and fibrosis. S100A4 is upregulated in proliferative diabetic retinopathy and is associated with angiogenesis and fibrosis markers (33). The major pathological process in type 2 diabetes (T2DM) is pancreatic dysfunction, characterized by impaired insulin secretion (34). In advanced T2DM patients, abnormal ECM accumulation, or fibrosis, is frequently observed, which can lead to organ dysfunction (34). Studies combining bioinformatics analysis and animal models have shown that pancreatic inflammation and fibrosis contribute to the development of pancreatic dysfunction in T2DM patients (35). Renal fibrosis is common in various kidney diseases and leads to mechanical and electrical dysfunction, as well as the progression of renal failure (36). In the early stages of kidney stone formation or crystal-induced kidney damage, renal tubular epithelial cells (TEC) undergo epithelial-to-mesenchymal transition (EMT), leading to renal fibrosis (37). S100A4 is considered an inducer of the EMT process and has been widely reported in kidney fibrosis (27, 28). These findings highlight the critical role of S100A4 signaling in TEC during kidney regeneration and suggest its potential as a therapeutic target for initiating intrinsic repair processes in chronic and acute kidney diseases.

The human actin-related protein 2/3 complex (Arp2/3) is essential for actin filament branching and contains two ARPC1 subunits, with ARPC1B being significantly expressed in blood cells (38). Deletion of ARPC1B results in platelet abnormalities and triggers inflammatory diseases (38, 39). Moreover, almost complete loss of ARPC1B expression leads to platelet defects, including microthrombus formation and spreading defects, as well as eczematous lesions, leukocyte fragmentation, vasculitis, eosinophilia, and increased IgA and IgE levels (38). Increased IgE levels are involved in multiple immune pathways, including enhanced Th2 cytokine production (40). Clinical studies have found that perioperative Th2 dominance is associated with a higher risk of febrile urinary tract infections in patients undergoing ureteroscopic surgery for ureteral stones (41). The Th1/Th2 ratio is significantly correlated with impaired glucose homeostasis, abnormal lipid profiles, and insulin resistance in type 2 diabetes (42). In addition, the Th1/Th2 ratio plays an essential role in type 1 diabetes (43, 44). ARPC1B’s effects on diabetes are not limited to immune responses; studies have shown that ARPC1B is a substrate of PAK1 during mitosis (45), and changes in PAK1 levels regulate the tissue crosstalk of pancreatic β cells, affecting systemic glucose homeostasis (46). These insights emphasize the potential novel link between ARPC1B regulation of kidney stones and diabetes metabolism.

The transcription factor CCAAT/enhancer-binding protein delta (CEBPD) belongs to the CCAAT/enhancer-binding protein family. It plays functional roles in cell differentiation, movement, growth arrest, cell death, metabolism, ECM generation, and immune responses (47–50). Research has shown that CEBPD can promote macrophage aggregation by activating FN-1 expression, and inhibiting CEBPD prevents kidney ischemia-reperfusion injury. CEBPD expression can induce upregulation of CHOP expression, leading to pro-apoptotic endoplasmic reticulum stress (51). Endoplasmic reticulum stress-mediated cell apoptosis can contribute to calcium oxalate stone formation (52). Additionally, IL-17 levels increase in kidney damage caused by calcium oxalate crystallization (53), and CEBPD, as an IL-17 downstream effector gene, is activated to perform its biological function (54, 55). The role of CEBPD in diabetes is less well understood, but it has been shown that during type 1 diabetes, pro-inflammatory cytokines (such as IL-1β, IFN-γ, and TNF-α) secreted by infiltrating immune cells alter the expression of key gene networks in β cells, resulting in local inflammation and β cell apoptosis. CEBPD has anti-apoptotic and anti-inflammatory effects in pancreatic β cells (56). These findings suggest that the biological processes related to S100A4, ARPC1B, and CEBPD may synergistically regulate the pathophysiology of kidney stones and diabetes. 

**Figure 10:**
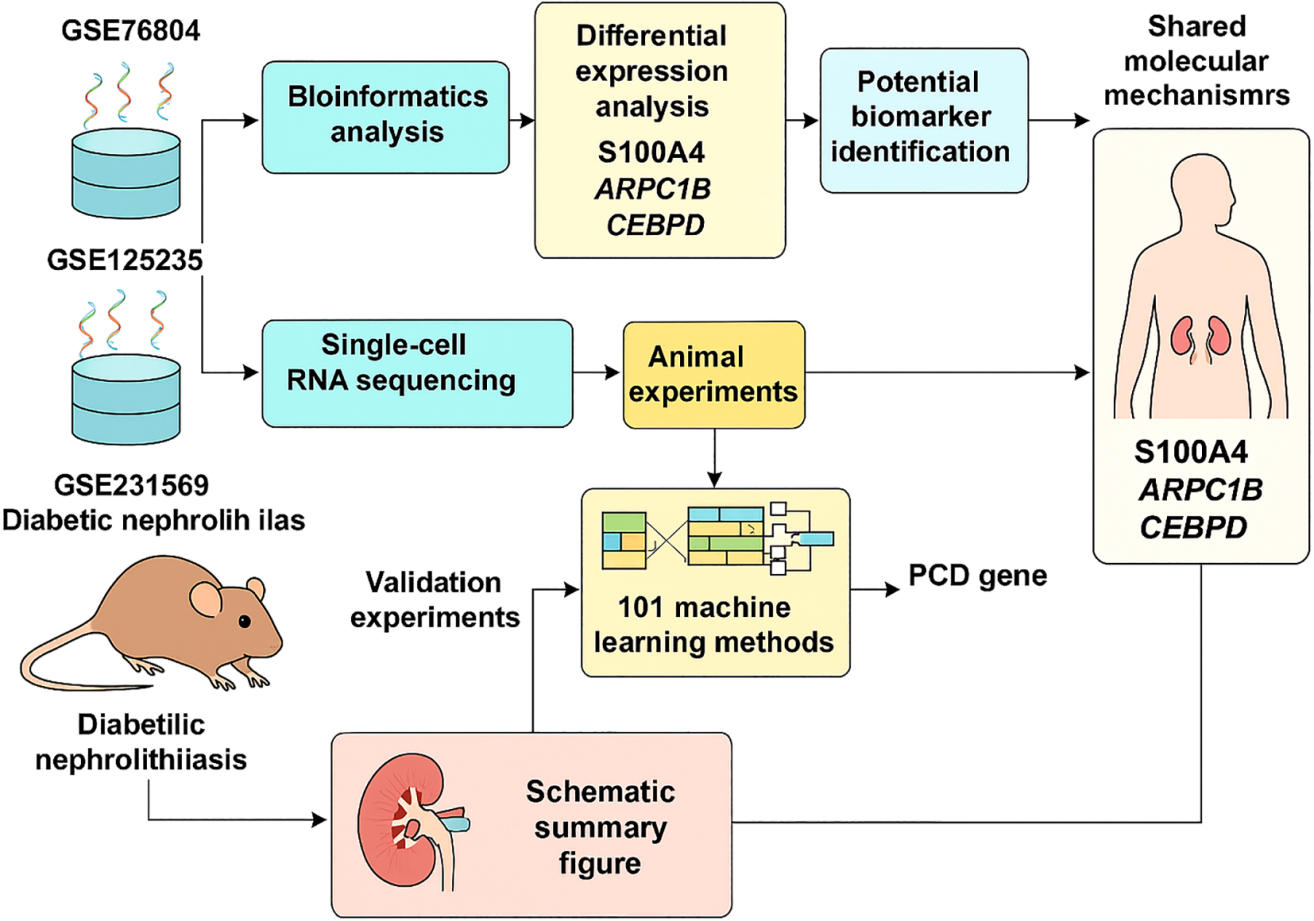
Flowchart depicting research methodology on diabetic nephrolithiasis.

As shown in **Figure 10**, the overall workflow of the study integrates single-cell transcriptomic analysis, bulk RNA-seq, machine learning screening, and *in vivo* validation to uncover shared molecular signatures between diabetes and nephrolithiasis. Overall, our study suggests that S100A4, ARPC1B, and CEBPD may serve as valuable biomarkers that play a crucial role in the shared pathogenesis of kidney stones and diabetes. However, our study has some limitations. The current results emphasize the need for prospective clinical trials to further validate the clinical applicability of our findings. The mechanisms of S100A4, ARPC1B, and CEBPD in kidney stones and diabetes should be further explored in wet lab experiments. Lastly, we used correction algorithms to mitigate these differences, but the cohorts included still have differences in scale and sequencing platforms.

In addition, although we validated the expression and functional relevance of the candidate biomarkers using a mouse model of diabetic nephrolithiasis, interspecies variation represents a non-negligible source of bias. Physiological and transcriptomic differences between mice and humans may affect the translatability of findings. While murine models offer mechanistic insights into disease progression, further functional validation using human tissues or patient-derived samples is essential to enhance the clinical relevance of the results.

## 5 Conclusion

Our study has preliminarily identified unique biomarkers and pathways shared between kidney stones and diabetes at the transcriptomic level. By integrating different datasets and bioinformatics technologies, we have identified S100A4, ARPC1B, and CEBPD as core biomarkers in the common mechanisms of these diseases, making them promising therapeutic targets.

## Data Availability

The datasets presented in this study can be found in online repositories. The names of the repository/repositories and accession number(s) can be found in the article/supplementary material.

## Supplementary material

Supplementary Figure 1 Quality control filtering on the GSE231569 dataset

Supplementary Figure 2 Cell clusters and differential genes were identified, and KEGG and Reactome analyses were performed on the differential genes.

Supplementary Figure 3 Gene expression levels of 12 genes(EGR1, ATF3, AOC1, BTG2, JUN, RHOB, S100A4, GDF15, GADD45B, CEBPD, DDIT4, AR PC1B) in different groups.

Supplementary Figure 4 PPI network construction for 12 genes by STRING database and GENEMANIA database.

Supplementary Figure 5 Correlation analysis of the 3 hub genes with all genes using heatmaps to show the expression of positively correlated top50 genes, respectively.
