# Supplementary figures and images for: Leveraging the integration of bioinformatics and machine learning to uncover common biomarkers and molecular pathways underlying diabetes and nephrolithiasis
Source: Front Immunol. 2025 Jul 11;16:1574157. doi: 10.3389/fimmu.2025.1574157 (PMC12289493; doi:10.3389/fimmu.2025.1574157)

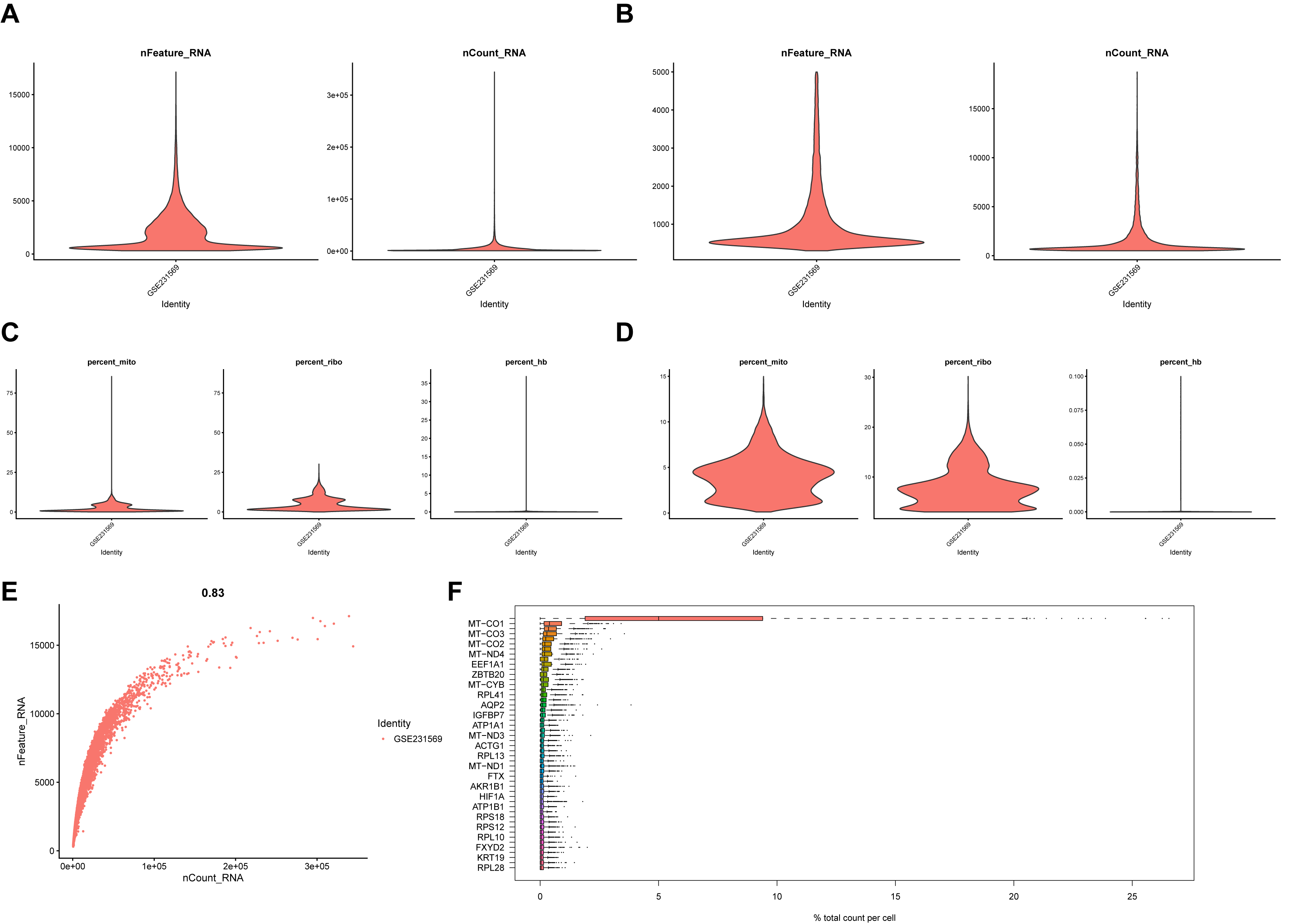

Supplement: Supplementary Figure 1 — Quality control filtering on the GSE231569 dataset [file Image1.tif]

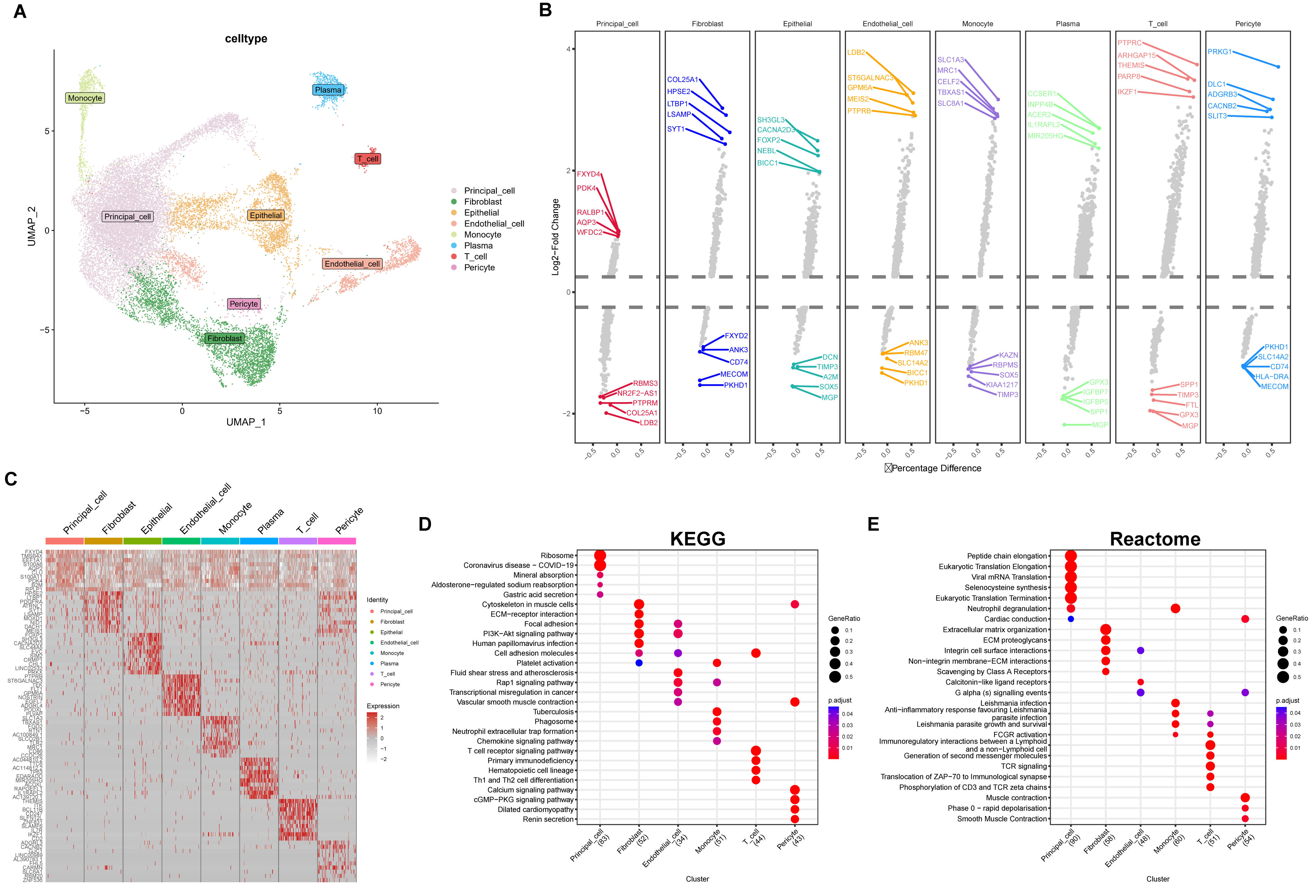

Supplement: Supplementary Figure 2 — Cell clusters and differential genes were identified, and KEGG and Reactome analyses were performed on the differential genes. [file Image2.tif]

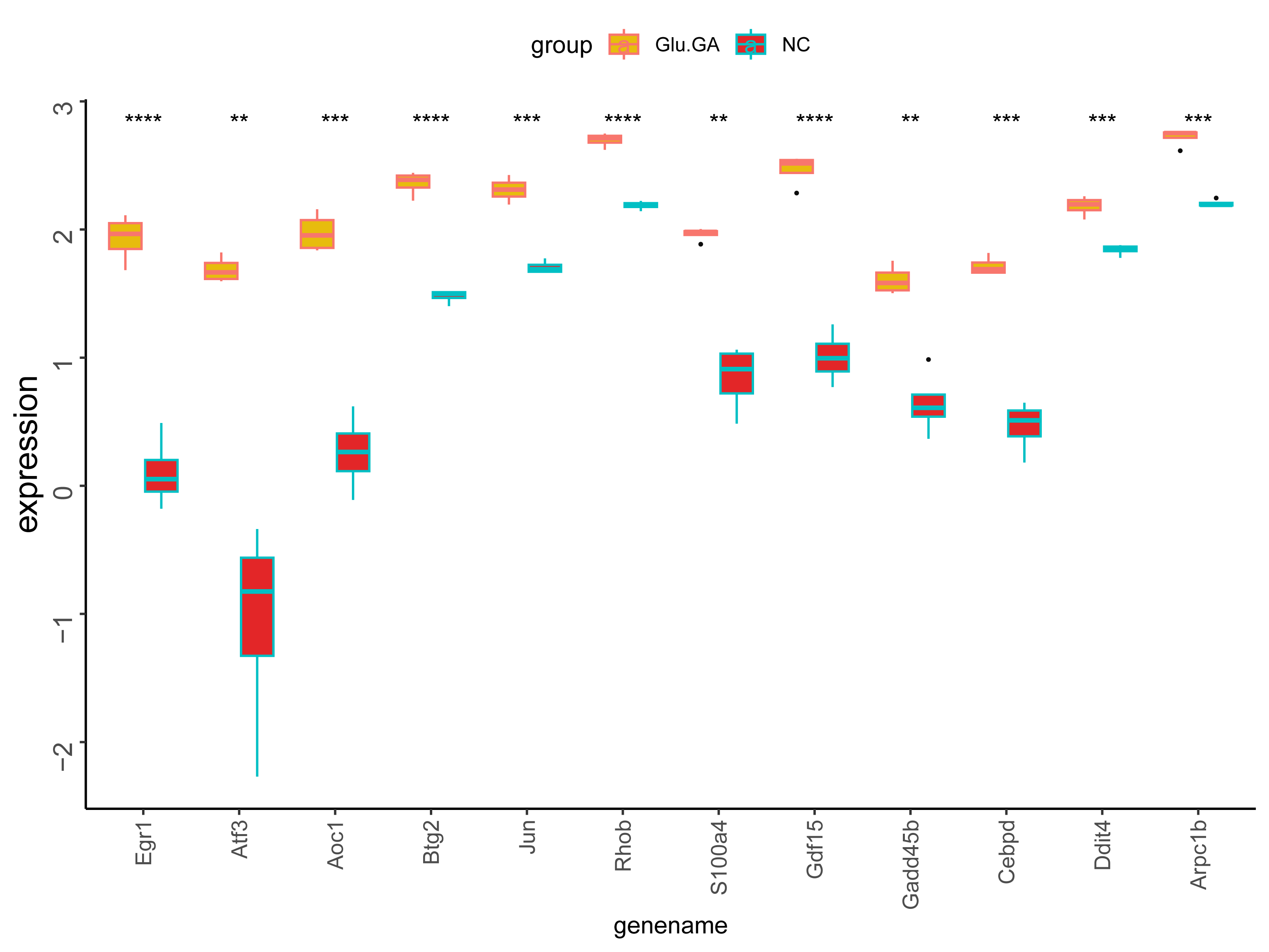

Supplement: Supplementary Figure 3 — Gene expression levels of 12 genes(EGR1, ATF3, AOC1, BTG2, JUN, RHOB, S100A4, GDF15, GADD45B, CEBPD, DDIT4, AR PC1B) in different groups. [file Image3.tif]

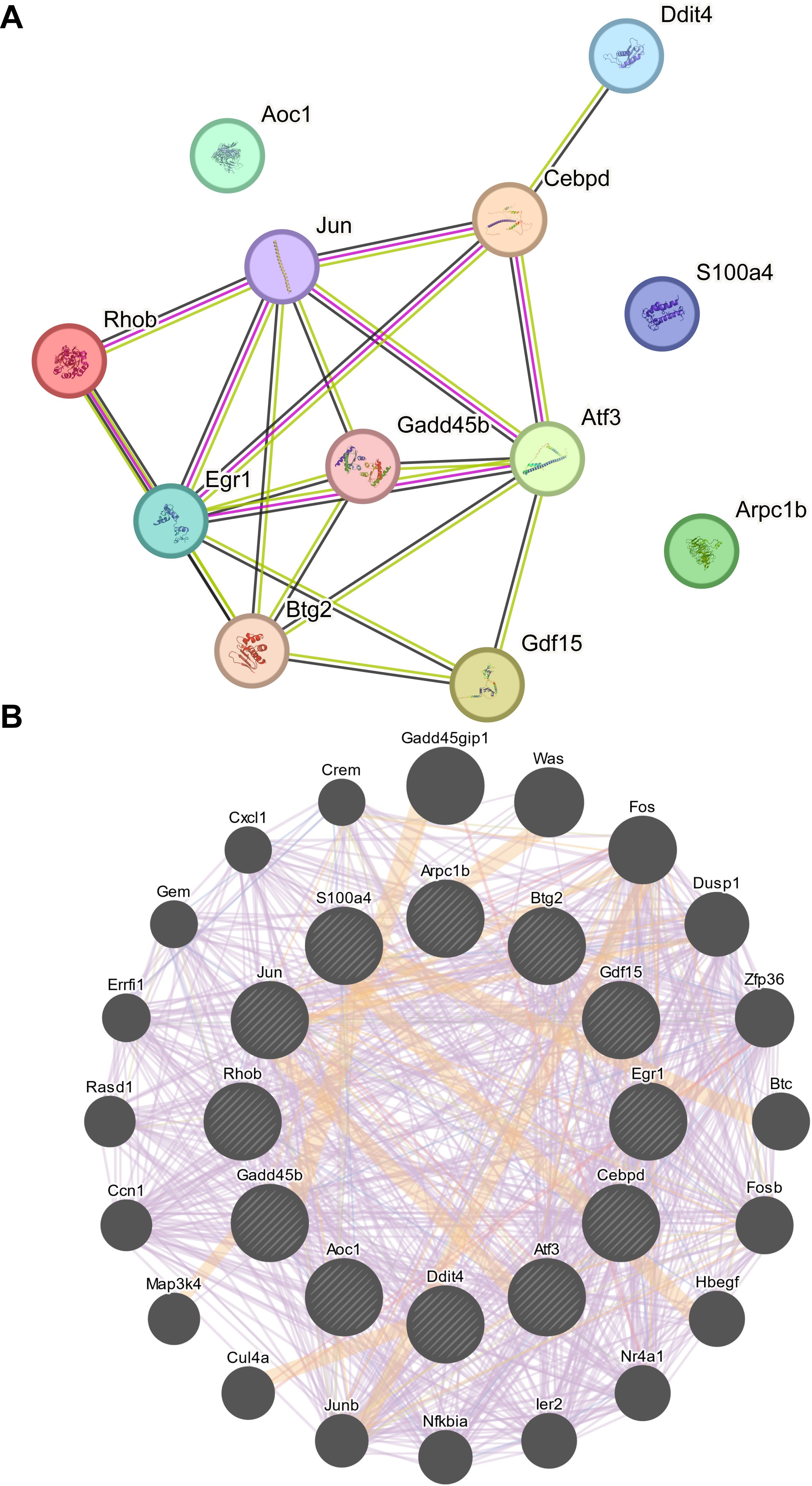

Supplement: Supplementary Figure 4 — PPI network construction for 12 genes by STRING database and GENEMANIA database. [file Image4.tif]

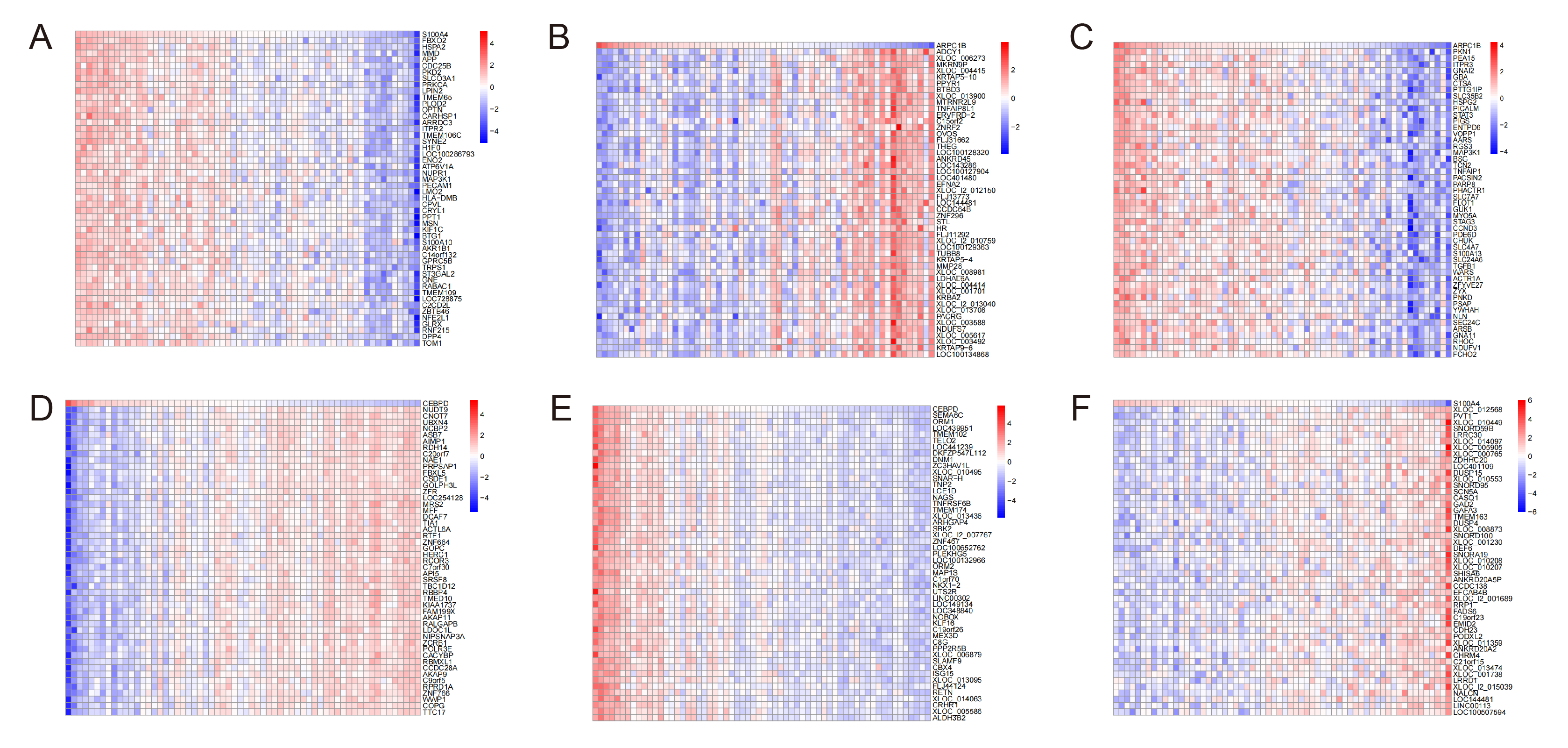

Supplement: Supplementary Figure 5 — Correlation analysis of the 3 hub genes with all genes using heatmaps to show the expression of positively correlated top50 genes, respectively. [file Image5.tif]
