# Supplementary material for: Leveraging the integration of bioinformatics and machine learning to uncover common biomarkers and molecular pathways underlying diabetes and nephrolithiasis
Source: Front Immunol. 2025 Jul 11;16:1574157. doi: 10.3389/fimmu.2025.1574157 (PMC12289493; doi:10.3389/fimmu.2025.1574157)
Supplement: Supplementary file 6 [file Table1.docx]

Supplementary Table 1. Genes included in different machine learning methods

| Gene | Features algorithm |
| --- | --- |
| S100A4 | Lasso+Stepglm[both] |
| CEBPD | Lasso+Stepglm[both] |
| ARPC1B | Lasso+Stepglm[both] |
| EGR1 | SVM |
| ATF3 | SVM |
| BTG2 | SVM |
| JUN | SVM |
| RHOB | SVM |
| S100A4 | SVM |
| GDF15 | SVM |
| GADD45B | SVM |
| CEBPD | SVM |
| DDIT4 | SVM |
| ARPC1B | SVM |
| RHOB | glmBoost+SVM |
| S100A4 | glmBoost+SVM |
| CEBPD | glmBoost+SVM |
| ARPC1B | glmBoost+SVM |
| EGR1 | Ridge |
| ATF3 | Ridge |
| BTG2 | Ridge |
| JUN | Ridge |
| RHOB | Ridge |
| S100A4 | Ridge |
| GDF15 | Ridge |
| GADD45B | Ridge |
| CEBPD | Ridge |
| DDIT4 | Ridge |
| ARPC1B | Ridge |
| EGR1 | Lasso+SVM |
| JUN | Lasso+SVM |
| RHOB | Lasso+SVM |
| S100A4 | Lasso+SVM |
| GDF15 | Lasso+SVM |
| CEBPD | Lasso+SVM |
| DDIT4 | Lasso+SVM |
| ARPC1B | Lasso+SVM |
| RHOB | glmBoost+Ridge |
| S100A4 | glmBoost+Ridge |
| CEBPD | glmBoost+Ridge |
| ARPC1B | glmBoost+Ridge |
| EGR1 | Enet[alpha=0.1] |
| JUN | Enet[alpha=0.1] |
| RHOB | Enet[alpha=0.1] |
| S100A4 | Enet[alpha=0.1] |
| GDF15 | Enet[alpha=0.1] |
| GADD45B | Enet[alpha=0.1] |
| CEBPD | Enet[alpha=0.1] |
| DDIT4 | Enet[alpha=0.1] |
| ARPC1B | Enet[alpha=0.1] |
| RHOB | glmBoost+Enet[alpha=0.1] |
| S100A4 | glmBoost+Enet[alpha=0.1] |
| CEBPD | glmBoost+Enet[alpha=0.1] |
| ARPC1B | glmBoost+Enet[alpha=0.1] |
| EGR1 | Enet[alpha=0.2] |
| RHOB | Enet[alpha=0.2] |
| S100A4 | Enet[alpha=0.2] |
| GDF15 | Enet[alpha=0.2] |
| CEBPD | Enet[alpha=0.2] |
| DDIT4 | Enet[alpha=0.2] |
| ARPC1B | Enet[alpha=0.2] |
| EGR1 | Enet[alpha=0.3] |
| RHOB | Enet[alpha=0.3] |
| S100A4 | Enet[alpha=0.3] |
| GDF15 | Enet[alpha=0.3] |
| CEBPD | Enet[alpha=0.3] |
| DDIT4 | Enet[alpha=0.3] |
| ARPC1B | Enet[alpha=0.3] |
| RHOB | glmBoost+Enet[alpha=0.3] |
| S100A4 | glmBoost+Enet[alpha=0.3] |
| CEBPD | glmBoost+Enet[alpha=0.3] |
| ARPC1B | glmBoost+Enet[alpha=0.3] |
| RHOB | glmBoost+Enet[alpha=0.2] |
| S100A4 | glmBoost+Enet[alpha=0.2] |
| CEBPD | glmBoost+Enet[alpha=0.2] |
| ARPC1B | glmBoost+Enet[alpha=0.2] |
| RHOB | Enet[alpha=0.4] |
| S100A4 | Enet[alpha=0.4] |
| GDF15 | Enet[alpha=0.4] |
| CEBPD | Enet[alpha=0.4] |
| DDIT4 | Enet[alpha=0.4] |
| ARPC1B | Enet[alpha=0.4] |
| RHOB | glmBoost+Enet[alpha=0.4] |
| S100A4 | glmBoost+Enet[alpha=0.4] |
| CEBPD | glmBoost+Enet[alpha=0.4] |
| ARPC1B | glmBoost+Enet[alpha=0.4] |
| RHOB | Lasso+glmBoost |
| S100A4 | Lasso+glmBoost |
| CEBPD | Lasso+glmBoost |
| ARPC1B | Lasso+glmBoost |
| RHOB | Enet[alpha=0.5] |
| S100A4 | Enet[alpha=0.5] |
| CEBPD | Enet[alpha=0.5] |
| ARPC1B | Enet[alpha=0.5] |
| RHOB | glmBoost |
| S100A4 | glmBoost |
| CEBPD | glmBoost |
| ARPC1B | glmBoost |
| RHOB | glmBoost+Enet[alpha=0.5] |
| S100A4 | glmBoost+Enet[alpha=0.5] |
| CEBPD | glmBoost+Enet[alpha=0.5] |
| ARPC1B | glmBoost+Enet[alpha=0.5] |
| S100A4 | Enet[alpha=0.6] |
| CEBPD | Enet[alpha=0.6] |
| ARPC1B | Enet[alpha=0.6] |
| RHOB | glmBoost+Enet[alpha=0.6] |
| S100A4 | glmBoost+Enet[alpha=0.6] |
| CEBPD | glmBoost+Enet[alpha=0.6] |
| ARPC1B | glmBoost+Enet[alpha=0.6] |
| RHOB | glmBoost+Enet[alpha=0.7] |
| S100A4 | glmBoost+Enet[alpha=0.7] |
| CEBPD | glmBoost+Enet[alpha=0.7] |
| ARPC1B | glmBoost+Enet[alpha=0.7] |
| RHOB | glmBoost+Enet[alpha=0.8] |
| S100A4 | glmBoost+Enet[alpha=0.8] |
| CEBPD | glmBoost+Enet[alpha=0.8] |
| ARPC1B | glmBoost+Enet[alpha=0.8] |
| RHOB | Enet[alpha=0.8] |
| S100A4 | Enet[alpha=0.8] |
| CEBPD | Enet[alpha=0.8] |
| ARPC1B | Enet[alpha=0.8] |
| S100A4 | Enet[alpha=0.9] |
| CEBPD | Enet[alpha=0.9] |
| ARPC1B | Enet[alpha=0.9] |
| EGR1 | Lasso |
| RHOB | Lasso |
| S100A4 | Lasso |
| GDF15 | Lasso |
| CEBPD | Lasso |
| DDIT4 | Lasso |
| ARPC1B | Lasso |
| RHOB | Enet[alpha=0.7] |
| S100A4 | Enet[alpha=0.7] |
| CEBPD | Enet[alpha=0.7] |
| ARPC1B | Enet[alpha=0.7] |
| RHOB | glmBoost+Enet[alpha=0.9] |
| S100A4 | glmBoost+Enet[alpha=0.9] |
| CEBPD | glmBoost+Enet[alpha=0.9] |
| ARPC1B | glmBoost+Enet[alpha=0.9] |
| RHOB | glmBoost+Lasso |
| S100A4 | glmBoost+Lasso |
| CEBPD | glmBoost+Lasso |
| ARPC1B | glmBoost+Lasso |
| EGR1 | Lasso+plsRglm |
| JUN | Lasso+plsRglm |
| RHOB | Lasso+plsRglm |
| S100A4 | Lasso+plsRglm |
| GDF15 | Lasso+plsRglm |
| CEBPD | Lasso+plsRglm |
| DDIT4 | Lasso+plsRglm |
| ARPC1B | Lasso+plsRglm |
| RHOB | glmBoost+plsRglm |
| S100A4 | glmBoost+plsRglm |
| CEBPD | glmBoost+plsRglm |
| ARPC1B | glmBoost+plsRglm |
| RHOB | glmBoost+Stepglm[forward] |
| S100A4 | glmBoost+Stepglm[forward] |
| CEBPD | glmBoost+Stepglm[forward] |
| ARPC1B | glmBoost+Stepglm[forward] |
| EGR1 | Lasso+Stepglm[forward] |
| JUN | Lasso+Stepglm[forward] |
| RHOB | Lasso+Stepglm[forward] |
| S100A4 | Lasso+Stepglm[forward] |
| GDF15 | Lasso+Stepglm[forward] |
| CEBPD | Lasso+Stepglm[forward] |
| DDIT4 | Lasso+Stepglm[forward] |
| ARPC1B | Lasso+Stepglm[forward] |
| S100A4 | RF+SVM |
| ARPC1B | RF+SVM |
| EGR1 | RF+SVM |
| BTG2 | RF+SVM |
| RHOB | RF+SVM |
| EGR1 | Stepglm[forward] |
| ATF3 | Stepglm[forward] |
| BTG2 | Stepglm[forward] |
| JUN | Stepglm[forward] |
| RHOB | Stepglm[forward] |
| S100A4 | Stepglm[forward] |
| GDF15 | Stepglm[forward] |
| GADD45B | Stepglm[forward] |
| CEBPD | Stepglm[forward] |
| DDIT4 | Stepglm[forward] |
| ARPC1B | Stepglm[forward] |
| EGR1 | plsRglm |
| ATF3 | plsRglm |
| BTG2 | plsRglm |
| JUN | plsRglm |
| RHOB | plsRglm |
| S100A4 | plsRglm |
| GDF15 | plsRglm |
| GADD45B | plsRglm |
| CEBPD | plsRglm |
| DDIT4 | plsRglm |
| ARPC1B | plsRglm |
| S100A4 | RF+Ridge |
| ARPC1B | RF+Ridge |
| EGR1 | RF+Ridge |
| BTG2 | RF+Ridge |
| RHOB | RF+Ridge |
| S100A4 | RF+Enet[alpha=0.1] |
| ARPC1B | RF+Enet[alpha=0.1] |
| EGR1 | RF+Enet[alpha=0.1] |
| BTG2 | RF+Enet[alpha=0.1] |
| RHOB | RF+Enet[alpha=0.1] |
| S100A4 | RF+Stepglm[forward] |
| ARPC1B | RF+Stepglm[forward] |
| EGR1 | RF+Stepglm[forward] |
| BTG2 | RF+Stepglm[forward] |
| RHOB | RF+Stepglm[forward] |
| S100A4 | RF+Enet[alpha=0.2] |
| ARPC1B | RF+Enet[alpha=0.2] |
| EGR1 | RF+Enet[alpha=0.2] |
| BTG2 | RF+Enet[alpha=0.2] |
| RHOB | RF+Enet[alpha=0.2] |
| S100A4 | RF+Enet[alpha=0.3] |
| ARPC1B | RF+Enet[alpha=0.3] |
| RHOB | RF+Enet[alpha=0.3] |
| S100A4 | RF+Enet[alpha=0.6] |
| ARPC1B | RF+Enet[alpha=0.6] |
| RHOB | RF+Enet[alpha=0.6] |
| S100A4 | RF+Lasso |
| ARPC1B | RF+Lasso |
| EGR1 | RF+Lasso |
| BTG2 | RF+Lasso |
| RHOB | RF+Lasso |
| S100A4 | RF+Enet[alpha=0.5] |
| ARPC1B | RF+Enet[alpha=0.5] |
| RHOB | RF+Enet[alpha=0.5] |
| S100A4 | RF+glmBoost |
| ARPC1B | RF+glmBoost |
| EGR1 | RF+glmBoost |
| RHOB | RF+glmBoost |
| S100A4 | RF+Stepglm[both] |
| EGR1 | RF+Stepglm[both] |
| RHOB | RF+Stepglm[both] |
| S100A4 | RF+Stepglm[backward] |
| EGR1 | RF+Stepglm[backward] |
| RHOB | RF+Stepglm[backward] |
| S100A4 | Stepglm[both]+Ridge |
| CEBPD | Stepglm[both]+Ridge |
| ARPC1B | Stepglm[both]+Ridge |
| S100A4 | Stepglm[backward]+Ridge |
| CEBPD | Stepglm[backward]+Ridge |
| ARPC1B | Stepglm[backward]+Ridge |
| S100A4 | Stepglm[both]+plsRglm |
| CEBPD | Stepglm[both]+plsRglm |
| ARPC1B | Stepglm[both]+plsRglm |
| S100A4 | Stepglm[backward]+plsRglm |
| CEBPD | Stepglm[backward]+plsRglm |
| ARPC1B | Stepglm[backward]+plsRglm |
| S100A4 | Stepglm[both]+Enet[alpha=0.9] |
| CEBPD | Stepglm[both]+Enet[alpha=0.9] |
| ARPC1B | Stepglm[both]+Enet[alpha=0.9] |
| S100A4 | Stepglm[backward]+Enet[alpha=0.9] |
| CEBPD | Stepglm[backward]+Enet[alpha=0.9] |
| ARPC1B | Stepglm[backward]+Enet[alpha=0.9] |
| S100A4 | Stepglm[both]+Enet[alpha=0.1] |
| CEBPD | Stepglm[both]+Enet[alpha=0.1] |
| ARPC1B | Stepglm[both]+Enet[alpha=0.1] |
| S100A4 | Stepglm[backward]+Enet[alpha=0.1] |
| CEBPD | Stepglm[backward]+Enet[alpha=0.1] |
| ARPC1B | Stepglm[backward]+Enet[alpha=0.1] |
| S100A4 | Stepglm[both]+Enet[alpha=0.8] |
| CEBPD | Stepglm[both]+Enet[alpha=0.8] |
| ARPC1B | Stepglm[both]+Enet[alpha=0.8] |
| S100A4 | Stepglm[backward]+Enet[alpha=0.8] |
| CEBPD | Stepglm[backward]+Enet[alpha=0.8] |
| ARPC1B | Stepglm[backward]+Enet[alpha=0.8] |
| S100A4 | Stepglm[both]+Enet[alpha=0.2] |
| CEBPD | Stepglm[both]+Enet[alpha=0.2] |
| ARPC1B | Stepglm[both]+Enet[alpha=0.2] |
| S100A4 | Stepglm[backward]+Enet[alpha=0.2] |
| CEBPD | Stepglm[backward]+Enet[alpha=0.2] |
| ARPC1B | Stepglm[backward]+Enet[alpha=0.2] |
| S100A4 | Stepglm[both]+Lasso |
| CEBPD | Stepglm[both]+Lasso |
| ARPC1B | Stepglm[both]+Lasso |
| S100A4 | Stepglm[backward]+Lasso |
| CEBPD | Stepglm[backward]+Lasso |
| ARPC1B | Stepglm[backward]+Lasso |
| S100A4 | Stepglm[both]+Enet[alpha=0.6] |
| CEBPD | Stepglm[both]+Enet[alpha=0.6] |
| ARPC1B | Stepglm[both]+Enet[alpha=0.6] |
| S100A4 | Stepglm[backward]+Enet[alpha=0.6] |
| CEBPD | Stepglm[backward]+Enet[alpha=0.6] |
| ARPC1B | Stepglm[backward]+Enet[alpha=0.6] |
| S100A4 | glmBoost+GBM |
| ARPC1B | glmBoost+GBM |
| CEBPD | glmBoost+GBM |
| RHOB | glmBoost+GBM |
| S100A4 | Stepglm[both]+Enet[alpha=0.7] |
| CEBPD | Stepglm[both]+Enet[alpha=0.7] |
| ARPC1B | Stepglm[both]+Enet[alpha=0.7] |
| S100A4 | Stepglm[backward]+Enet[alpha=0.7] |
| CEBPD | Stepglm[backward]+Enet[alpha=0.7] |
| ARPC1B | Stepglm[backward]+Enet[alpha=0.7] |
| S100A4 | Lasso+Stepglm[backward] |
| CEBPD | Lasso+Stepglm[backward] |
| ARPC1B | Lasso+Stepglm[backward] |
| S100A4 | Stepglm[both] |
| CEBPD | Stepglm[both] |
| ARPC1B | Stepglm[both] |
| S100A4 | Stepglm[backward] |
| CEBPD | Stepglm[backward] |
| ARPC1B | Stepglm[backward] |
| S100A4 | glmBoost+Stepglm[both] |
| CEBPD | glmBoost+Stepglm[both] |
| ARPC1B | glmBoost+Stepglm[both] |
| S100A4 | glmBoost+Stepglm[backward] |
| CEBPD | glmBoost+Stepglm[backward] |
| ARPC1B | glmBoost+Stepglm[backward] |
| S100A4 | Stepglm[both]+Enet[alpha=0.4] |
| CEBPD | Stepglm[both]+Enet[alpha=0.4] |
| ARPC1B | Stepglm[both]+Enet[alpha=0.4] |
| S100A4 | Stepglm[backward]+Enet[alpha=0.4] |
| CEBPD | Stepglm[backward]+Enet[alpha=0.4] |
| ARPC1B | Stepglm[backward]+Enet[alpha=0.4] |
| S100A4 | Stepglm[both]+Enet[alpha=0.3] |
| CEBPD | Stepglm[both]+Enet[alpha=0.3] |
| ARPC1B | Stepglm[both]+Enet[alpha=0.3] |
| S100A4 | Stepglm[backward]+Enet[alpha=0.3] |
| CEBPD | Stepglm[backward]+Enet[alpha=0.3] |
| ARPC1B | Stepglm[backward]+Enet[alpha=0.3] |
| S100A4 | Stepglm[both]+glmBoost |
| CEBPD | Stepglm[both]+glmBoost |
| ARPC1B | Stepglm[both]+glmBoost |
| S100A4 | Stepglm[backward]+glmBoost |
| CEBPD | Stepglm[backward]+glmBoost |
| ARPC1B | Stepglm[backward]+glmBoost |
| S100A4 | Stepglm[both]+Enet[alpha=0.5] |
| CEBPD | Stepglm[both]+Enet[alpha=0.5] |
| ARPC1B | Stepglm[both]+Enet[alpha=0.5] |
| S100A4 | Stepglm[backward]+Enet[alpha=0.5] |
| CEBPD | Stepglm[backward]+Enet[alpha=0.5] |
| ARPC1B | Stepglm[backward]+Enet[alpha=0.5] |
| S100A4 | glmBoost+RF |
| ARPC1B | glmBoost+RF |
| CEBPD | glmBoost+RF |
| S100A4 | RF |
| ARPC1B | RF |
| JUN | RF |
| RHOB | RF |
| GADD45B | RF |
| S100A4 | Lasso+GBM |
| ARPC1B | Lasso+GBM |
| DDIT4 | Lasso+GBM |
| CEBPD | Lasso+GBM |
| EGR1 | Lasso+GBM |
| JUN | Lasso+GBM |
| GDF15 | Lasso+GBM |
| RHOB | Lasso+GBM |
| S100A4 | RF+GBM |
| ARPC1B | RF+GBM |
| RHOB | RF+GBM |
| EGR1 | RF+GBM |
| BTG2 | RF+GBM |
| S100A4 | GBM |
| ARPC1B | GBM |
| CEBPD | GBM |
| DDIT4 | GBM |
| ATF3 | GBM |
| EGR1 | GBM |
| GDF15 | GBM |
| RHOB | GBM |
| JUN | GBM |
| GADD45B | GBM |
| BTG2 | GBM |
| S100A4 | Stepglm[both]+SVM |
| CEBPD | Stepglm[both]+SVM |
| ARPC1B | Stepglm[both]+SVM |
| S100A4 | Stepglm[backward]+SVM |
| CEBPD | Stepglm[backward]+SVM |
| ARPC1B | Stepglm[backward]+SVM |
| S100A4 | Lasso+RF |
| ARPC1B | Lasso+RF |
| EGR1 | Lasso+RF |
| S100A4 | Stepglm[both]+GBM |
| ARPC1B | Stepglm[both]+GBM |
| CEBPD | Stepglm[both]+GBM |
| S100A4 | Stepglm[backward]+GBM |
| ARPC1B | Stepglm[backward]+GBM |
| CEBPD | Stepglm[backward]+GBM |
| EGR1 | LDA |
| ATF3 | LDA |
| BTG2 | LDA |
| JUN | LDA |
| RHOB | LDA |
| S100A4 | LDA |
| GDF15 | LDA |
| GADD45B | LDA |
| CEBPD | LDA |
| DDIT4 | LDA |
| ARPC1B | LDA |
| RHOB | glmBoost+LDA |
| S100A4 | glmBoost+LDA |
| CEBPD | glmBoost+LDA |
| ARPC1B | glmBoost+LDA |
| S100A4 | RF+LDA |
| ARPC1B | RF+LDA |
| EGR1 | RF+LDA |
| BTG2 | RF+LDA |
| RHOB | RF+LDA |
| S100A4 | Stepglm[both]+LDA |
| CEBPD | Stepglm[both]+LDA |
| ARPC1B | Stepglm[both]+LDA |
| S100A4 | Stepglm[backward]+LDA |
| CEBPD | Stepglm[backward]+LDA |
| ARPC1B | Stepglm[backward]+LDA |
| EGR1 | Lasso+LDA |
| JUN | Lasso+LDA |
| RHOB | Lasso+LDA |
| S100A4 | Lasso+LDA |
| GDF15 | Lasso+LDA |
| CEBPD | Lasso+LDA |
| DDIT4 | Lasso+LDA |
| ARPC1B | Lasso+LDA |
| EGR1 | XGBoost |
| ATF3 | XGBoost |
| BTG2 | XGBoost |
| JUN | XGBoost |
| RHOB | XGBoost |
| S100A4 | XGBoost |
| GDF15 | XGBoost |
| GADD45B | XGBoost |
| CEBPD | XGBoost |
| DDIT4 | XGBoost |
| ARPC1B | XGBoost |
| EGR1 | Lasso+XGBoost |
| JUN | Lasso+XGBoost |
| RHOB | Lasso+XGBoost |
| S100A4 | Lasso+XGBoost |
| GDF15 | Lasso+XGBoost |
| CEBPD | Lasso+XGBoost |
| DDIT4 | Lasso+XGBoost |
| ARPC1B | Lasso+XGBoost |
| RHOB | glmBoost+XGBoost |
| S100A4 | glmBoost+XGBoost |
| CEBPD | glmBoost+XGBoost |
| ARPC1B | glmBoost+XGBoost |
| S100A4 | RF+XGBoost |
| ARPC1B | RF+XGBoost |
| EGR1 | RF+XGBoost |
| BTG2 | RF+XGBoost |
| RHOB | RF+XGBoost |
| S100A4 | Stepglm[both]+XGBoost |
| CEBPD | Stepglm[both]+XGBoost |
| ARPC1B | Stepglm[both]+XGBoost |
| S100A4 | Stepglm[backward]+XGBoost |
| CEBPD | Stepglm[backward]+XGBoost |
| ARPC1B | Stepglm[backward]+XGBoost |
| EGR1 | NaiveBayes |
| ATF3 | NaiveBayes |
| BTG2 | NaiveBayes |
| JUN | NaiveBayes |
| RHOB | NaiveBayes |
| S100A4 | NaiveBayes |
| GDF15 | NaiveBayes |
| GADD45B | NaiveBayes |
| CEBPD | NaiveBayes |
| DDIT4 | NaiveBayes |
| ARPC1B | NaiveBayes |
| EGR1 | Lasso+NaiveBayes |
| JUN | Lasso+NaiveBayes |
| RHOB | Lasso+NaiveBayes |
| S100A4 | Lasso+NaiveBayes |
| GDF15 | Lasso+NaiveBayes |
| CEBPD | Lasso+NaiveBayes |
| DDIT4 | Lasso+NaiveBayes |
| ARPC1B | Lasso+NaiveBayes |
| RHOB | glmBoost+NaiveBayes |
| S100A4 | glmBoost+NaiveBayes |
| CEBPD | glmBoost+NaiveBayes |
| ARPC1B | glmBoost+NaiveBayes |
| S100A4 | RF+NaiveBayes |
| ARPC1B | RF+NaiveBayes |
| EGR1 | RF+NaiveBayes |
| BTG2 | RF+NaiveBayes |
| RHOB | RF+NaiveBayes |
| S100A4 | Stepglm[both]+NaiveBayes |
| CEBPD | Stepglm[both]+NaiveBayes |
| ARPC1B | Stepglm[both]+NaiveBayes |
| S100A4 | Stepglm[backward]+NaiveBayes |
| CEBPD | Stepglm[backward]+NaiveBayes |
| ARPC1B | Stepglm[backward]+NaiveBayes |
